# Activation of anionic redox in d^0^ transition metal chalcogenides by anion doping

**DOI:** 10.1038/s41467-021-25760-8

**Published:** 2021-09-16

**Authors:** Bernhard T. Leube, Clara Robert, Dominique Foix, Benjamin Porcheron, Remi Dedryvère, Gwenaëlle Rousse, Elodie Salager, Pierre-Etienne Cabelguen, Artem M. Abakumov, Hervé Vezin, Marie-Liesse Doublet, Jean-Marie Tarascon

**Affiliations:** 1 Collège de France, Chaire de Chimie du Solide et de l’Energie, UMR 8260, 11 Place Marcelin Berthelot, 75231 Cedex 05, Paris, France; 2 Réseau sur le Stockage Electrochimique de l’Energie (RS2E), FR CNRS 3459, 33 Rue Saint Leu, 80039 Amiens, France; 3 ICGM, Univ Montpellier, CNRS, ENSCM, Montpellier, France; 4 IPREM/ECP (UMR 5254), Université de Pau, 2 Avenue Pierre Angot, 64053 Pau, Cedex 9 France; 5 CNRS, CEMHTI UPR3079, Université d’Orléans, 1D avenue de la recherche scientifique, 45071 Cedex 2, Orléans, France; 6 Sorbonne Université, 4 Place Jussieu, 75005 Paris, France; 7 Umicore, New Business Incubation, 31 rue Marais, 1000 Brussels, Belgium; 8 Center for Energy Science and Technology, Skolkovo Institute of Science and Technology, Nobel str. 3, 121205 Moscow, Russia; 9 Univ. Lille, UMR CNRS 8516 LASIRE,, F-59000 Lille, France

**Keywords:** Batteries, Materials for energy and catalysis

## Abstract

Expanding the chemical space for designing novel anionic redox materials from oxides to sulfides has enabled to better apprehend fundamental aspects dealing with cationic-anionic relative band positioning. Pursuing with chalcogenides, but deviating from cationic substitution, we here present another twist to our band positioning strategy that relies on mixed ligands with the synthesis of the Li_2_TiS_3-x_Se_x_ solid solution series. Through the series the electrochemical activity displays a bell shape variation that peaks at 260 mAh/g for the composition *x* = 0.6 with barely no capacity for the *x* = 0 and *x* = 3 end members. We show that this capacity results from cumulated anionic (Se^2−^/Se^n−^) and (S^2−^/S^n−^) and cationic Ti^3+^/Ti^4+^ redox processes and provide evidence for a metal-ligand charge transfer by temperature-driven electron localization. Moreover, DFT calculations reveal that an anionic redox process cannot take place without the dynamic involvement of the transition metal electronic states. These insights can guide the rational synthesis of other Li-rich chalcogenides that are of interest for the development of solid-state batteries.

## Abstract (web summary)

Activation of anionic redox in battery materials promises great benefits for battery materials, but remains an elusive phenomenon. Here, the authors present anion-doping as a novel strategy to unlock electrochemical activity in the cobalt/nickel free cathode material, Li_2_TiS_3-x_Se_x_.

## Introduction

Layered lithium-rich transition metal (*TM*) oxides, Li_*x*_*TM*_*y*_O_2_ (*x* > *y*), constitute a promising family for high-capacity cathode materials, relying on cationic and anionic redox processes for charge compensation^1,2^. The archetypical Li_1.2_Ni_0.13_Mn_0.54_Co_0.13_O_2_ (Li-rich NMC) can deliver specific capacities above 270 mAh/g to reach 1.000 Wh/kg of specific energy at the material level^3,4^. As established by extensive theoretical work, the introduction of alkaline ions into the TM layer results in 2*p* lone pairs on the oxygen^5–7^. Their electronic states serve as a reservoir of electrons that can potentially participate in anionic redox processes and liberate additional capacity compared to conventional cathode materials based on cationic redox, provided that they are made accessible through *TM*(*nd*)/O(2*p*) hybridization owing to local distortions (see Supplementary Figure 1a). In the majority of Li-rich materials, however, the formation of holes on oxygen leads to unstable electronic configurations, which is evidenced by structural rearrangements such as phase transitions, cationic migration, or oxygen release upon oxidation. In consequence, exploitation of anionic redox in battery materials is hampered by irreversible capacities, sluggish kinetics, large hysteresis, and voltage fade^8^. These effects can be mitigated in materials based on TMs with low-lying *d*-orbital levels such as Li_2_IrO_3_ or Li_2_RuO_3_^9,10^. In these systems, the unstable electronic configurations of oxidized oxygen anions are stabilized by strong interactions with the *TM d* orbitals. Among the different interpretations proposed in the literature for this oxygen hole stabilization, both the formation of (O–O)^*n*^^−^ species^9^ or the formation of Ir = O double bonds involve the O 2*p* lone pairs^11^. Due to difficulties in decoupling the anionic and cationic processes in highly covalent systems, it is likely that both mechanisms contribute almost equally to the electronic structure of the delithiated materials as different mesomeric structures.

In this context, chalcogenide (*Ch* = S, Se, and Te)-based materials are attractive since anion–cation redox competition was recognized in such materials back to the 1960s by Rouxel^12^. Ongoing from O to S, Se, or Te, the top of the anionic band is raised and can eventually interact strongly with the *d* states of TMs. Internal redox chemistry can be triggered by careful selection of the chemical elements and consecutive alignment of the respective bands: TiS_2_ is a semiconductor (Ti^4+^), while TiSe_2_ is a semimetal^13^, and finally TiTe_2_ is a metal with a significant transfer of electrons from the anionic Te band into the cationic bands of Ti^14^, as corroborated by density functional theory (DFT) calculations^15^. The propensity of stabilizing isolated holes at the top of the anion band increases concomitantly from O to Te: it is not possible to remove copper from Cu_0.5_Cr^3+^S_2_, while the formation of the selenide homolog Cr^3+^Se_2_ is feasible^16^. Equally, this explains why when moving from the left-hand side of the periodic table the disulfides form layered structures with fully reduced S^2−^ anions (TiS_2_, NbS_2_), while the formation of persulfide type S–S bonds is encountered only on the right-hand side of the periodic table (e.g., marcasite/pyrite type Fe^2+^S_2_, Co^2+^S_2_, and Ni^2+^S_2_)^17,18^. Ultimately, the persulfide TiS_3_ shows two types of oxidation states for the sulfur (S^2−^ and (S_2_)^2−^)^19,20^, which suggests that anionic redox can be activated in materials with d^0^ metals. These mechanisms of hole stabilization constitute a rich playground for the synthesis of materials with reversible anionic redox chemistry^21^. Within this framework, NaCrS_2_ is a notable material, as it is one of the first layered chalcogenides to solely utilize the S^2−^/S^*n*−^ redox couple upon electrochemical cycling^22^. Furthermore, anion doping has been reported for Li_2_FeS_2 − *x*_Se_*x*_ in an attempt to control the covalency and the corresponding anionic redox potential^23^. Unfortunately, the overall performance of this material deteriorated with increasing Se content.

Pursuing this direction, researchers targeted the electrochemically inactive Li_2_TiS_3_ phase, structurally analogous to Li-rich oxides Li_2_MnO_3_, because of its high theoretical capacity (339 mAh/g)^24^. The introduction of non-d^0^ cations (Ti^3+^, Fe^2+^, and Co^2+^)^24–26^ associated with the concomitant lowering of the cationic *d* levels (e.g., M^3+/4+^ bands just above the S 3*p* bands) is a viable strategy to activate Li_2_TiS_3_ using either solid-state synthesis in quartz tubes or ball milling^27^. In this work, we propose an alternative approach to electrochemical activate Li_2_TiS_3_: instead of lowering the cationic levels, we adjust the energy and symmetry of the anionic *p* levels by doping Se^2−^ into Li_2_TiS_3 − *x*_Se_*x*_ (Supplementary Figure 1b). We demonstrate that this strategy triggers reversible electrochemical activity in Li-rich d^0^ *TM* chalcogenides. An arsenal of complementary methods (diffraction, electrochemistry, X-ray photoemission spectroscopy (XPS), electron paramagnetic resonance (EPR) spectroscopy, and nuclear magnetic resonance (NMR) spectroscopy, DFT calculations) is used to understand and explain the underlying mechanisms of anionic redox in these mixed anionic materials. Altogether, these results point towards better exploitation of the ligand chemistry to broaden the class of electrode materials benefiting from anionic redox activity.

## Results

**Fig. 1:**
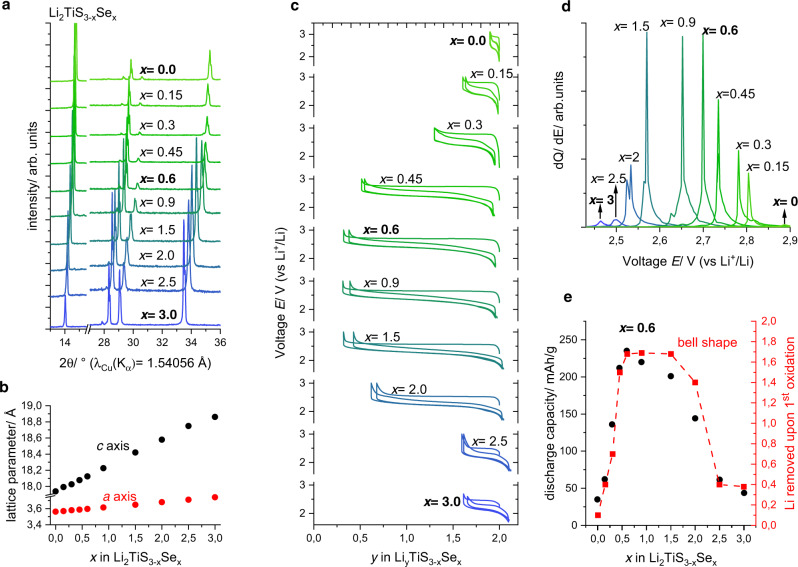
Structural and electrochemical characterization of Li_2_TiS_3 − *x*_Se_*x*_. **a** PXRD patterns of synthesized materials in Li_2_TiS_3 − *x*_Se_*x*_: a shift towards lower 2*θ* angles (higher *d*-spacing) is observed upon Se introduction. **b** Lattice parameters refined in $R\bar{3}m$ plotted against the Se content *x* in Li_2_TiS_3 − *x*_Se_*x*_. **c** Voltage traces of the first two galvanostatic cycles. **d** Change of the differential capacity d*Q*/d*E* of the first oxidation process with Se doping. The lowering of the activation potential indicates successful manipulation of the anionic band. The decreased voltage is consistent with increased covalency of Ti–Se bonds compared to Ti–S bonds and with the lower chemical hardness of Se compared to S. **e** Gravimetric discharge capacity of the first discharge process (black) and the electrochemical activity (amount of removable Li upon the first oxidation) (red).

The synthetic conditions reported for Li_2_TiS_3_ were used as a starting point for preparing members of the Li_2_TiS_3 − *x*_Se_*x*_ series (*x* = 0.15, 0.3, 0.45, 0.6, 0.9, 1.5, 2.0, 2.5, and 3)^24^. For compositions *x* ≤ 2, Li_2_S, TiS_2_, and TiSe_2_ were used as reagents, while for compositions *x* > 2, Li_2_S, Li_2_Se, and TiSe_2_ were ground and calcined in evacuated quartz tubes (see Supplementary information for details). Inductively coupled plasma analysis of the Li and Ti content of the final products confirmed that the ratio is constant along the full range 0 ≤ *x* ≤ 3. The powder XRD (PXRD) patterns of the as-synthesized materials (Fig. 1a) can be fully indexed to the monoclinic $C2/m$ space group or by disregarding superstructure reflections to the rhombohedral $R\bar{3}m$ space group. For simplicity of comparison, the corresponding unit-cell dimensions were refined in $R\bar{3}m$ (Fig. 1b). The lattice parameters increase linearly throughout the solid solution due to the larger anionic radius of Se^2−^ (1.98 Å) compared to S^2−^ (1.848 Å)^28^.

To interrogate the impact of the anion substitution in Li_2_TiS_3 − *x*_Se_*x*_ on the electrochemistry, Swagelok-type cells were assembled and cycled against Li^+^/Li as shown in Fig. 1c. Altogether, the electrochemical activity follows a bell-shaped curve with *x*: while at the end members Li_2_TiS_3_ and Li_2_TiSe_3_ allow for the removal of only 0.1 and 0.4 Li, respectively; up to 1.7 Li can be extracted at 0.6 < *x* < 1.5, see Fig. 1e (red). For all members of the Li_2_TiS_3 − *x*_Se_*x*_ series, the first charging process is characterized by a voltage plateau alike what has been reported in other Li-rich *TM* chalcogenides utilizing anionic redox^24,25,29,30^. The potential of this plateau decreases along Li_2_TiS_3 − *x*_Se_*x*_ from 2.9 V in Li_2_TiS_3_ to 2.46 V in Li_2_TiSe_3_, as shown in Fig. 1d. Upon discharge all the removed Li can be reinserted in Li_*y*_TiS_3 − *x*_Se_*x*_, resulting in S-shaped voltage traces. However, the smaller hysteresis together with the absence of irreversibility during the first cycle suggests much limited irreversible structural damages for the heavier chalcogenides S/Se as compared to oxides. Altogether, the gravimetric discharge capacity peaks at *x* = 0.6 corresponding to a capacity of 235 mAh/g or 550 Wh/kg (Fig. 1e). It is worth noting that we could trigger an additional capacity of ca. 30 mAh/g (17%) by preparing the electrodes by ball milling, but such a benefit was negated by a poor cycling performance (see Supplementary Figure 2).

**Fig. 2:**
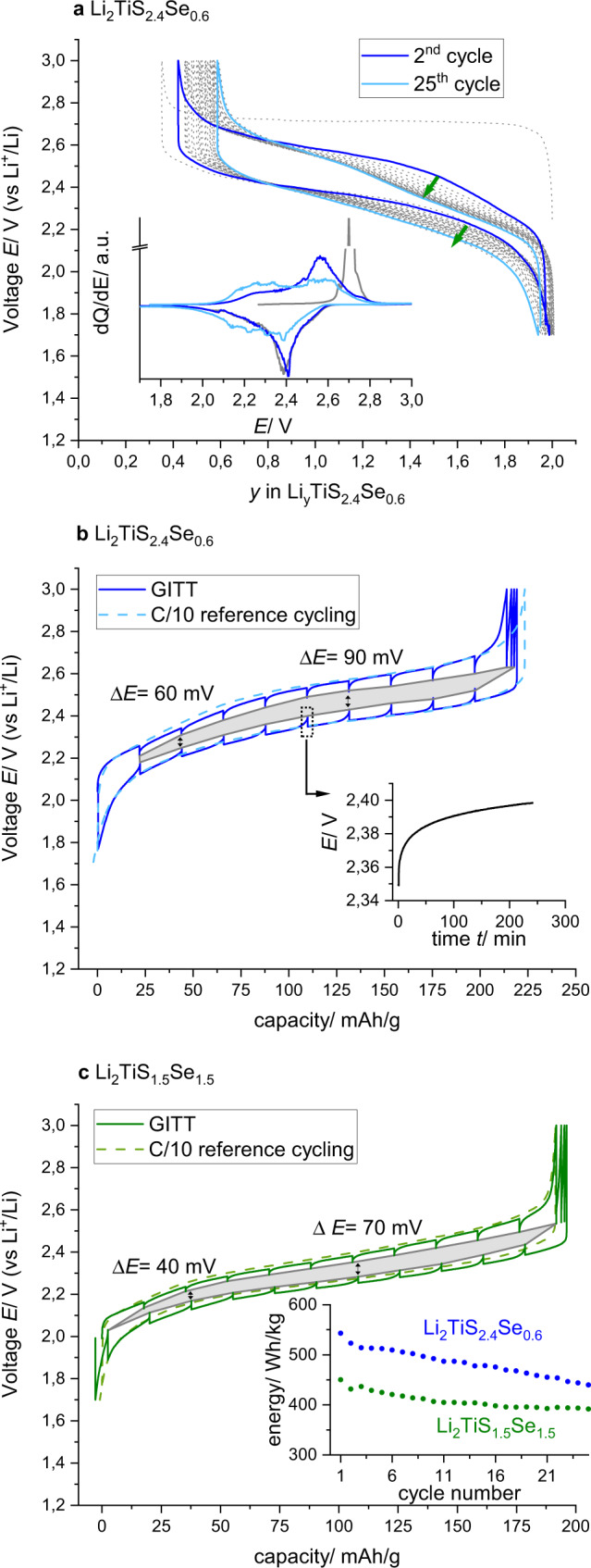
Electrochemistry of Li_2_TiS_2.4_Se_0.6_ and Li_2_TiS_1.5_Se_1.5_. **a** Voltage trace of Li_2_TiS_2.4_Se_0.6_ during 25 cycles (discharge-voltage drops from 2.33 to 2.25 V), inset: d*Q*/d*E* vs *E* for the 1st, 2nd and 25th cycle. **b** GITT experiment of Li_2_TiS_2.4_Se_0.6_. **c** GITT experiment of Li_2_TiS_2.4_Se_0.6_, inset: comparison of discharge energy of Li_2_TiS_2.4_Se_0.6_ with Li_2_TiS_1.5_Se_1.5_ during the first 25 cycles.

Galvanostatic cycling of Li/Li_2_TiS_2.4_Se_0.6_ (*x* = 0.6) cells over 25 cycles shows sustained capacity retention with, however, a noticeable discharge-voltage fade of 80 mV (Fig. 2(a)). This is comparable to a voltage drop of Δ*E* = 50 mV in LTFS (Li_1.13_Ti_0.57_Fe_0.3_S_2_)^25^, but dramatically lower than ~200 mV reported for Li-rich oxides^31^. The differential capacity d*Q*/d*E* is given for the 1st, 2nd, and 25th cycles in the inset. After the initial activation plateau at 2.7 V, consecutive oxidation cycles display broad contributions with two maxima at 2.3 and 2.6 V, respectively. This is reminiscent of Li_2_TiS_3_, for which an oxidation process at ca. 2.3 V was related to the cationic Ti^3+/4+^ redox couple and a second process at 2.6 V to an anionic redox process S^2−/*n*−^ (*n* < 2)^24^.

To check the effect of increasing the Se content on both the polarization and anionic redox response, galvanostatic intermittent titration technique experiments were conducted on Li_2_TiS_2.4_Se_0.6_ and Li_2_TiS_1.5_Se_1.5_. The voltage gap (Fig. 2b, c) measured at the same Li content (*y* = 1) after 4 h of relaxation amounts to Δ*E* = 90 mV for *x* = 0.6 as compared to 70 mV for *x* = 1.5. This voltage gap could not be mitigated by applying a very low cycling *C*/40 rate (compare to Supplementary Figure 3a, b), implying that the system is kinetically confined in a metastable pathway. To get further insights on the origins of this sluggish kinetics, we decided to progressively open the charge window in for the *x* = 0.6 sample (see Supplementary Figure 3c). Interestingly, the charge profiles are identical, but the discharge profiles steadily fall along with simultaneous growth of polarization overpotential after the delithiation level crosses the mark of 20% of SoC (i.e., 50 mAh/g with applied voltages *E* between 2.4 and 2.5 V). These results imply the existence of two electrochemical processes having different kinetics as frequently observed for anionic redox materials, with faster and slower ones being associated with the cationic and anionic redox couples, respectively. Altogether, we note that increasing the selenium content to *x* = 1.5 (Li_2_TiS_1.5_Se_1.5_) partially mitigates the hysteresis most likely due to the increase of covalence in the Ti–*Ch* bonds, which enables faster charge transfer. However, this positive effect is counterbalanced at high Se content by a lowering of electrochemical capacity, hence questioning the role of the crystal vs electronic structure in governing the performances of the Li_2_TiS_3 − *x*_Se_*x*_ members.

**Fig. 3:**
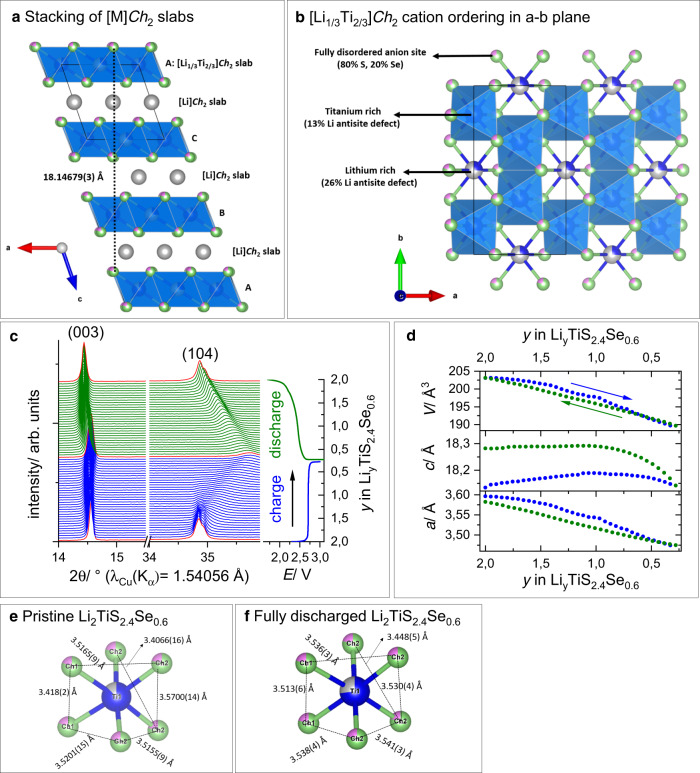
Crystal structure of Li_*y*_TiS_2.4_Se_0.6_. **a** Structure of Li_2_TiS_2.4_Se_0.6_ shown along the stacking axis: Li and Ti occupy all octahedral interstices present in the cubic close packing of Ch^2−^ anions. Partial ordering of the cations leads to the formation of [Li]*Ch*_2_ and [Li_1/3_Ti_2/3_]*Ch*_2_ layers. **b** Representation of the mixed [Li_1/3_Ti_2/3_]*Ch*_2_ slab: the partial ordering of Li vs Ti. **c** Evolution of the PXRD patterns of Li_*y*_TiS_2.4_Se_0.6_ during the 1st cycle of an operando PXRD experiment. **d** Corresponding evolution of the lattice parameters of Li_*y*_TiS_2.4_Se_0.6_ (space group: $R\bar{3}m$). **e**, **f** Local bonding environments around Ti1 of pristine Li_2_TiS_2.4_Se_0.6_ and fully discharged Li_2_TiS_2.4_Se_0.6_ (color code: blue: Ti, gray: Li, green: S, pink: Se).

Due to its superior energy density, Li_2_TiS_2.4_Se_0.6_ was studied in greater detail for its composition, morphology, structure and charge compensating mechanism, and coupling diffraction techniques with microscopy and spectroscopy. EDX compositional maps confirm the homogeneous distribution of Ti, S, and Se, in the atomic ratio 1.05(2):2.34(5):0.62(6), which is in excellent agreement with the nominal composition (Supplementary Figure 4). Electron diffraction (ED, Supplementary Figure 5) patterns reveal, in agreement with synchrotron XRD (SXRD), an O3 structure that can be indexed with the $R\bar{3}m$ unit cell (*a* ≈ 3.5 Å, *c* ≈ 18.2 Å). Additional sharp diffuse intensity lines clearly visible in the [110] ED pattern point towards a 2D honeycomb Li–Ti ordering and mandate a more rigorous crystallographic treatment in the monoclinic $C2/m$ space group. The appearance of this superstructure in the form of diffuse intensity rather than regular reflections points to the high concentration of stacking faults that is typical for this type of structure. The structural model of O3-type Li_2_TiS_3_ was used as a starting point for a combined Rietveld refinement of SXRD and neutron powder diffraction data^32^ in the $C2/m$ space group (Supplementary Figure 6 and Supplementary Table 1). The refined model confirms that Li_2_TiS_2.4_Se_0.6_ is isostructural to Li_2_TiS_3_ (Fig. 3a, b), with fully disordered S/Se anion sites and a large degree of disorder. Li–Ti antisite disorder (13%) of the intralayer honeycomb and stacking faults, stemming from differently oriented [Li_1/3_Ti_2/3_]*Ch*_2_ slabs, give rise to broad superstructure reflections at 3.5 Å <*d* < 5.5 Å. A [010] high-angle annular dark-field scanning transmission electron microscopy (HAADF-STEM) image nicely visualizes the O3 stacking of the closely packed layers along the *c*-axis, while the honeycomb ordering and stacking faults are clearly visible along the [110] direction (Supplementary Figure 7).

Finally, robust structural refinements were carried out on SXRD data of isostructural Li_2_TiS_1.5_Se_1.5_ and Li_2_TiSe_3_. These are based on the nominal composition, even though minute anionic or cationic vacancy concentration cannot be ruled out relying only on this set of data (Li_2_TiSe_3_ was refined in the $R\bar{3}m$ space group due to severe stacking faults) (Supplementary Figures 8 and 9 and Supplementary Tables 2 and 3). A compendium of selected metal–chalcogenide bond distances is given in Supplementary Table 4. With increasing Se doping in Li_2_TiS_3 − *x*_Se_*x*_, the Ti–*Ch* and Li–*Ch* bond lengths increase almost linearly in the pristine materials (from 2.5014 to 2.6137(4) Å).

The structural evolution of Li_*y*_TiS_2.4_Se_0.6_ induced by electrochemical cycling was studied in an *operando* PXRD experiment. Upon oxidation, a gradual shift of the (104) reflection towards smaller *d*-spacing (higher 2*θ* value) is observed until 1.7 Li are removed at 3.0 V (Fig. 3c). The delithiated material retains crystallinity albeit a broadening of the reflections, which is due to strain occurring in the individual particles upon Li removal. The PXRD pattern of the end-member Li_0.3_TiS_2.4_Se_0.6_ can be fully indexed to the $R\bar{3}m$ space group. On the subsequent discharge, a reverse peak shift is observed. The variation of the *a* and *c* unit-cell parameters of Li_*y*_TiS_2.4_Se_0.6_ through the first cycle is shown in Fig. 3d. During oxidation the unit cell contracts along the *a*-axis, while the *c*-axis shows non-monotonic changes: it expands until *y* = 1.1 and then shrinks during further delithiation. During discharge, the lattice parameter *a* expands linearly and reaches values close to the pristine material (3.5961(1) vs 3.5812(1) Å). Conversely, a nonlinear expansion occurs along *c* and the inter-slab distance in fully relithiated Li_2_TiS_2.4_Se_0.6_ is slightly larger than that in the pristine material (18.1315(10) vs 18.2638(4) Å). Upon subsequent charge–discharge cycles, a more reversible evolution is witnessed (Supplementary Figure 10).

To gain further structural insight into the Li uptake-removal process, a combination of ex situ diffraction experiments (SXRD, ED) and STEM imaging was performed on chemically charged Li_0.3_TiS_2.4_Se_0.6_ and fully discharged Li_2_TiS_2.4_Se_0.6_ (Supplementary Figures 5b, c and 11). Altogether, these techniques confirm the correct indexing to the rhombohedral $R\bar{3}m$ space group, while highlighting the suppression of the honeycomb ordering on charge. Strikingly, partial reconstruction of the honeycomb ordering in the [Li_1/3_Ti_2/3_]*Ch*_2_ slab is observed in the discharged sample by both ED (Supplementary Figure 5d) and SXRD refinement (Supplementary Figure 12 and Supplementary Table 5), albeit an increased Li–Ti antisite disorder (27%). Finally, HAADF-STEM images were collected on charged (Supplementary Figure 13) and discharged (Supplementary Figure 14) Li_*y*_TiS_2.4_Se_0.6_ after 40 electrochemical cycles to investigate the origin of the voltage fade upon increased cycling. However, no Ti migration from the [Li_1/3_Ti_2/3_]*Ch*_2_ slab into octahedral or tetrahedral interstices in the [Li]*Ch*_2_ slab was observed in either material. Similarly to the situation in LTFS, we find no experimental evidence of *Ch–Ch* dimer formation upon oxidation.

Although PXRD suggests the participation of the chalcogenide anions in the overall redox process, it cannot decouple how such a contribution is parted between S and Se. To answer this question, XPS was carried out on pristine materials (0 < *x* < 3) and on samples for *x* = 0.6 at different states of charge. The S 2*p*, Se 3*p*, and Ti 2*p* core spectra were collected at two different photon energies: 1487 eV (home XPS) and 10 keV (hard X-rays, i.e., HAXPES) to probe the sample surface (7–8 nm) and the bulk (43–44 nm), respectively^33^.

**Fig. 4:**
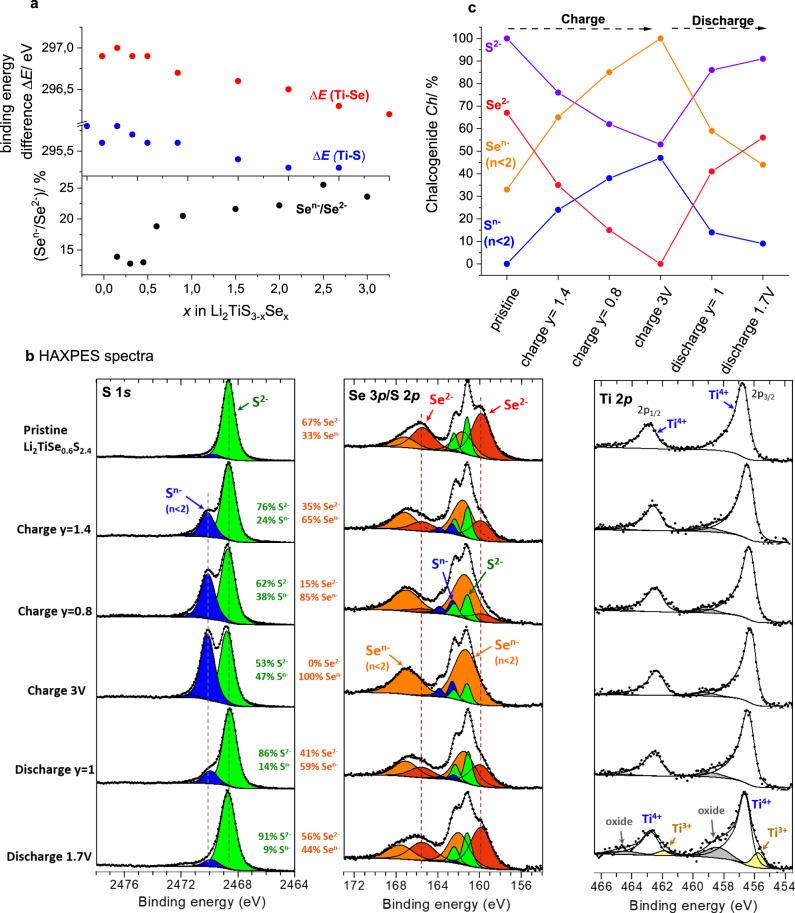
XPS/HAXPES experiments on Li_*y*_TiS_3 − *x*_Se_*x*_. **a** Binding energy difference Δ*E*(Ti–*Ch*) and the amount of partially Se^*n*−^ as a function of *x* in pristine Li_2_TiS_3 − *x*_Se_*x*_ as deduced from XPS data. **b** HAXPES S 1*s*, S 2*p/*Se 3*p* and Ti 2*p* spectra of Li_*y*_TiS_2.4_Se_0.6_ at different states of charge. **c** Proportion of reduced Ch^2−^ and partially oxidized Ch^*n*−^ in Li_*y*_TiS_2.4_Se_0.6_ at different states of charge as deduced from HAXPES spectra.

Starting with the pristine samples, we could deduce from the variation of the XPS spectra (Supplementary Figure 15) a decrease of the binding energy difference Δ*E*(Ti–*Ch*) between the Ti 2*p*_3/2_ and the chalcogenide S 2*p*_3/2_ or Se 3*p*_3/2_ peaks with increasing *x* (Fig. 4a). Concomitantly, there is an increase of oxidized Se^*n*−^, while S and Ti are barely sensitive to the Se content and remain as S^2−^ and Ti^4+^, respectively. Both trends are consistent with the increasingly covalent bonding when moving from S to Se, therefore implying less positively charged cations and less negatively charged anions. The increase of Ti–*Ch* bond covalency explains how charge balancing is assumed in these materials, whereas the Li/Ti ratio remains constant.

HAXPES and XPS spectra were then collected on ex situ samples of Li_*y*_TiS_2.4_Se_0.6_ (*x* = 0.6), recovered from electrochemical half-cells cycled to different states of charge (0.3 < *y* < 2, Supplementary Figure 16a). An inherent advantage of HAXPES within the scope of this study, besides its greater probing depth, relies on its feasibility to increase significantly the Se 3*p*/S 2*p* intensity ratio (multiplied by ∼4 compared to XPS), and also to access the S 1*s* core peak, which is free from any overlapping with selenium peak (Fig. 4b). This permits to decouple the S and Se contributions and quantify the oxidation states of both species as a function of *y*. The HAXPES S 1*s* and Ti 2*p* spectra confirm the presence of S^2−^ and Ti^4+^ in pristine Li_2_TiS_2.4_Se_0.6_ together with two forms of Se: fully reduced Se^2−^ (Se 3*p*_3/2_ at 159.9 eV) and partially oxidized Se^*n*−^ (Se 3*p*_3/2_ at 161.6 eV). Upon charge, there is a clear growth of the Se^*n*−^ (*n* < 2) component that is accompanied by the gradual disappearance of the signal assigned to Se^2−^ (Fig. 4b). Concomitantly, an additional S 1*s* component (∼2470 eV) corresponding to oxidized S species (S^*n−*^) emerges at higher binding energy. In the fully charged sample, selenium is predominantly oxidized to Se^*n*−^, while 50% of sulfur is found as S^*n*−^, implying that selenium is preferentially oxidized compared to sulfur. It is also important to notice the decrease of the binding energy difference between the Ti 2*p*_3/2_ component of Ti^4+^ and the S 2*p*_3/2_ component of sulfide S^2−^, Δ*E*(Ti–S), which drops from 295.6 to 295.1 eV, relating to the increasing covalent character of the Ti–S bond upon lithium extraction. This process is partially reversible upon the following discharge (increase up to 295.4 eV). It is also worth noting that the Se^*n*−^ peak is slightly shifting by −0.25 eV with respect to the Se^2−^ peak upon the charge, and is shifting back upon the following discharge. This shift is rather small but significant, which tends to indicate that the “*n*” value is not constant.

On subsequent discharge, oxidized sulfur (S^*n*−^) and selenium (Se^*n*−^) species are not fully re-reduced to S^2−^ and Se^2−^ (Fig. 4c). Astonishingly, in the Ti 2*p*_3/2_ core peak of the fully discharged sample, an additional peak at ∼455.8 eV appears that can be unambiguously ascribed to Ti^3+^, which integrates to ∼9 wt%. For better evidence of this additional peak, the derivative curve of Ti 2*p*_3/2_ component is shown in Supplementary Figure 16b. Consequently, once the activation process is achieved, titanium becomes part of the redox-compensation mechanism alike in Li-rich layered oxides. We further note a nearly constant proportion of the cationic and anionic redox species upon subsequent cycling (XPS data on 2nd cycle: see Supplementary Figure 17). Lastly, necessary mentioning is the presence of a well-defined peak at 459 eV in the XPS Ti 2*p* core spectrum for pristine Li_*y*_TiS_2.4_Se_0.6_ corresponding to the binding energy of Ti–O that is barely detectable in the HAXPES spectrum. Bearing in mind that HAXPES probes the sample in greater depth, this difference is indicative of minor amounts of superficial Ti–O species, as frequently reported for Ti-based sulfides.

Altogether, XPS/HAXPES enabled a decoupling of the S/Se redox processes while providing evidence for the triggering of Ti^4+/3+^ redox activity at the end of the first discharge. However, an intriguing result based on simple electroneutrality consideration regards the non-monotonous appearance of oxidized Se in the pristine samples while titanium remains apparently fully oxidized as Ti^4+^. To clarify this ambiguity, EPR spectroscopy was performed. EPR can detect the different contributions from the cations and anions to the electronic wavefunction and hence distinguishes between localized and delocalized electrons.

**Fig. 5:**
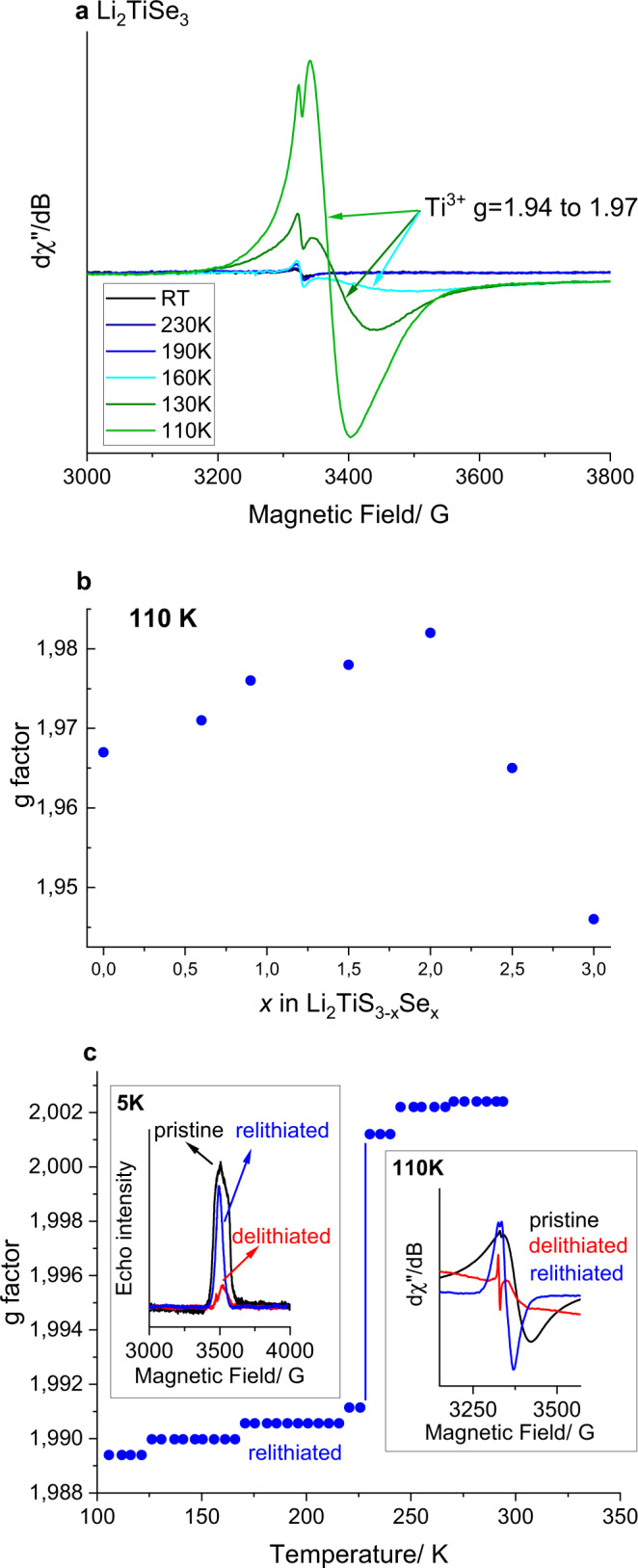
EPR results on Li_2_TiS_3 − *x*_Se_*x*_. **a** Temperature-dependent EPR spectra of Li_2_TiSe_3_ (**b**) evolution of the *g*-factor in Li_2_TiS_3 − *x*_Se_*x*_ at 110 K. **c** Main: temperature dependence of the *g*-factor of relithiated Li_*y*_TiS_2.4_Se_0.6_. Inset: EPR spectra of pristine Li_2_TiS_2.4_Se_0.6_ (black), delithiated Li_*y*_TiS_2.4_Se_0.6_ (red) and relithiated Li_*y*_TiS_2.4_Se_0.6_ (blue) at 5 and 110 K, respectively.

EPR spectra were measured for both pristine materials in Li_2_TiS_3 − *x*_Se_*x*_ and for chemically delithiated and relithiated Li_*y*_TiS_2.4_Se_0.6_ (for more information on synthesis see Supplementary information and Supplementary Figure 18). Regardless of the Se content, the EPR spectra of the pristine materials are nearly featureless at room temperature, suggesting, at first sight, the exclusive presence of Ti^4+^ (no *d* electrons). But signals associated with Ti^3+^ emerge at temperatures below ~170 K (Fig. 5a). The inability to detect Ti^3+^ at room temperature is not unusual as it commonly occurs in compounds within which Ti sits in perfectly octahedral environments so that the *T*_1e_ relaxation time is too short. A fully delocalized Ti^3+^/Ti^4+^ mixed-valence state associated with an average Ti^(4 − δ)+^ oxidation state with δ linked to the Se content is consistent with the absence of EPR signal at room temperature and with our inability to detect Ti^3+^ by XPS.

However, by lowering the temperature, the spin relaxation time of Ti^3+^ increases owing to the electron localization on Ti, which results in a progressive distortion of the octahedral site. Such a temperature-driven distortion is confirmed by the narrowing of the EPR signal linewidth leading to the appearance of separate and well-defined 3 eigenvalues for the *g*-factors at 5.2 K (Supplementary Figure 19) with the distortion being greater for Li_2_TiSe_3_ than for Li_2_TiS_3_.

Figure 5b shows the evolution of the *g*-factor in the pristine Li_2_TiS_3 − *x*_Se_*x*_ series deduced from the EPR spectra collected at 110 K. Interestingly, the g-factor does not vary linearly with x through this solid solution, but instead peaks near x = 1.5, which corresponds to a 50:50 S/Se composition. This can be understood as a superposition of TiS_6_, TiSe_6_, and mixed Ti(S/Se)_6_ octahedra: starting from the TiS_6_ end-member, the *g*-factor increases initially due to an increase of local structural disorder, or equivalently due to the widest variety of TiS_6 − *n*_Se_*n*_ environments (*n* = 1–6). Finally, the *g*-factor decreases due to the larger spin–orbit coupling of TiSe_6_ compared to TiS_6_. It is worth mentioning that pristine Li_2_TiSe_3_ shows a Pauli paramagnetic behavior (*χ* positive and independent of temperature), implying that Ti *d* and Se *p* states constitute its Fermi level.

Lastly, temperature-dependent EPR spectra were collected on chemically delithiated and relithiated samples of Li_*y*_TiS_2.4_Se_0.6_ (Fig. 5c), using I_2_ and *n*-BuLi as oxidizing and reducing agents, respectively. At room temperature, no Ti^3+^ is evidenced in neither sample. However, by lowering the temperature, we can track a change of the *g*-factor in the relithiated sample at around 225 K from values >2.0023 to smaller values, indicating localization of electrons on the Ti, and therefore validate the formation of Ti^3+^. Finally, low-temperature (5 K) EPR measurements using echo detection reveal a strong isotropic signal centered at *g* = 1.98 and an anisotropic signal (*g*_*x,y*_ = 1.97 and *g*_*z*_ = 1.95) for both the delithiated and relithiated sample, indicating the presence of Ti^3+^ (Fig. 5c (inset left)), with a higher Ti^3+^ content for the relithiated sample, as expected.

**Fig. 6:**
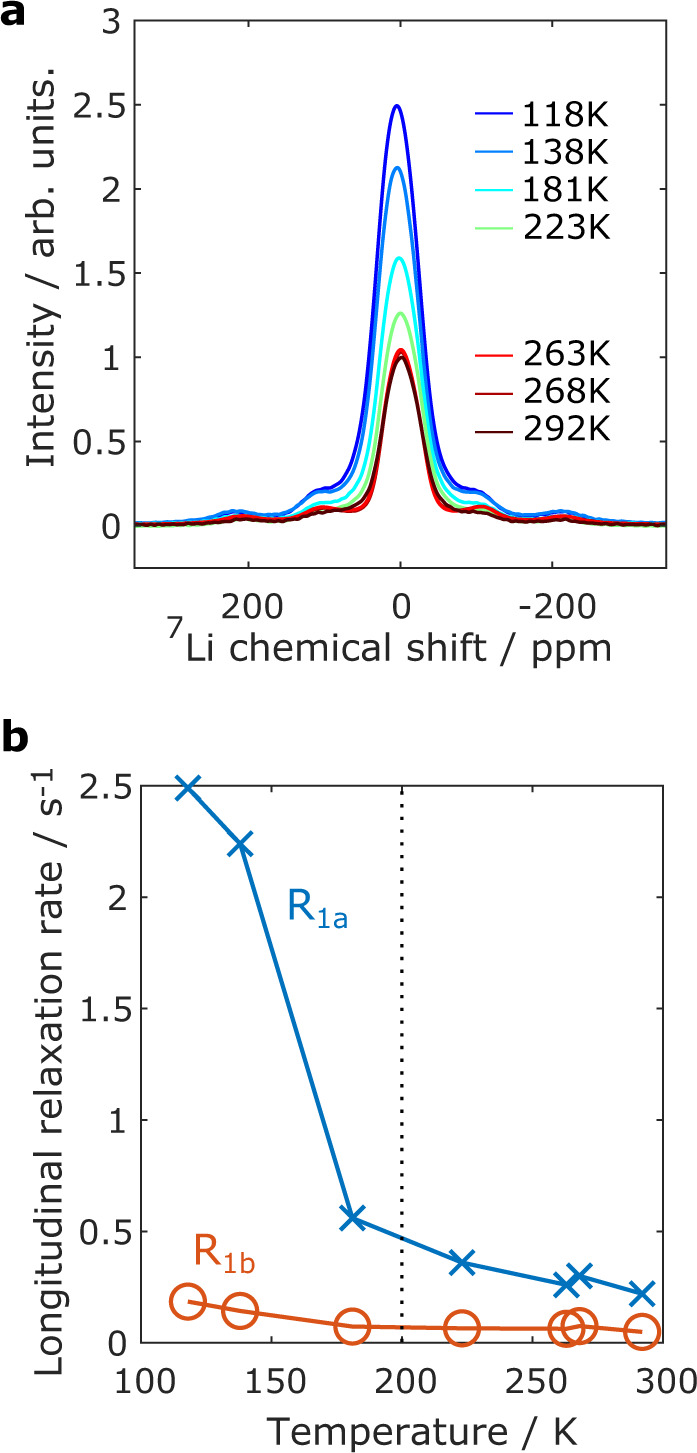
Solid-state NMR on Li_2_TiSe_3_. **a** Static ^7^Li spectra as a function of temperature. **b** ^7^Li longitudinal relaxation components *R*_1a_ and *R*_1b_ plotted against temperature.

To further interrogate this temperature-driven distortion deduced by EPR, we decided to complement our study by NMR, exploring the local ^77^Se and ^7^Li environments for the Li_2_TiSe_3_ sample in the temperature range from 292 to 118 K (see Supplementary information for details of fit). The static ^7^Li one-pulse NMR spectrum consists of a lineshape typical of quadrupolar nuclei with two sets of satellite transitions (Fig. 6a). The spectrum could be fitted reasonably with three components, in agreement with the well-resolved fast magic-angle spinning (MAS)-NMR ^7^Li NMR spectrum (Supplementary Figure 20): two peaks with a quadrupolar symmetric lineshape indicating anisotropic ^7^Li environments (*ν*_Q_ ~17 and ~38 kHz) and 1 gaussian peak of width ~50 ppm arising for more mobile or symmetric environments (Supplementary Table 7). The imperfections of the fit indicate a distribution in the quadrupolar environments, not quantified here.

Turning to the quadrupolar lineshapes of the ^7^Li one-pulse NMR spectra, they do not change significantly upon lowering of the temperature with changes in quadrupolar frequencies by a maximum 3 kHz, without any obvious trend. Interestingly, the area of the signal is rather constant at the higher temperatures then increases sharply below 200 K to reach 2.6 times the area of the spectrum recorded at 292 K (Supplementary Figure 21). This increase in area is accompanied by an increase of the chemical shift for the Gaussian peak, which reaches 5.8 ppm at 118 K. This shift, out of the typical range for Li species in a diamagnetic environment, together with the increased intensity, suggests the emergence of a new signal, which was probably broadened beyond detection at higher temperatures. In order to validate this hypothesis, ^7^Li longitudinal relaxation was measured at each temperature and fitted with two relaxation rate components (*R*_1a_ and *R*_1b_, Supplementary Table 8). Interestingly, while *R*_1b_ is small and did not vary much with temperature, *R*_1a_ shows a sharp increase when lowering the temperature below 200 K (Fig. 6b). This increase in relaxation rate when lowering temperature is unusual; the opposite would be expected from changes in the dynamics. It is assigned to a drastic change in the local electronic environment of the Li atoms, driven by unpaired electron density in the vicinity of the Li atoms. This is in agreement with the appearance of Ti^3+^ signature below 200 K in EPR due to a change in electronic relaxation. Similar measurements made on ^77^Se did not provide any variation in the measured chemical shift or longitudinal relaxation rates. This suggests that only the Se next to diamagnetic Ti^4+^ are detected, even at the lowest temperature reached here. The Se atoms next to the Ti^3+^ are still broadened beyond detection at 118 K, contrary to the ^7^Li atoms that are further away from the Ti atoms.

Altogether, our experimental results suggest that Se substitution in Li_2_TiS_3_ triggers charge transfer from Se to Ti. While oxidized Se^*n*−^ is clearly identified by XPS measurements, our complementary EPR study unambiguously shows that at room temperature the electrons transferred to the Ti *d* band are fully delocalized in the structure and therefore not detected in the Ti 2*p* XPS spectra. Finally, a combination of EPR and NMR experiments detects temperature-driven changes of relaxation phenomena associated with changes in the local electronic environment of Ti and Li in Li_2_TiSe_3_.

**Fig. 7:**
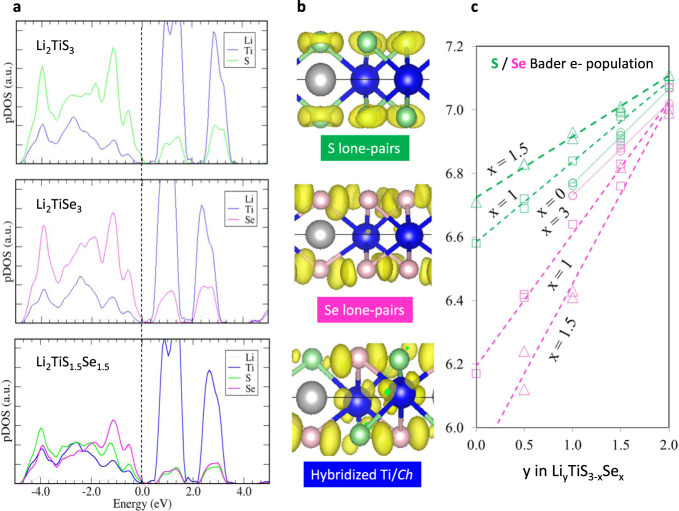
Theoretical calculations on Li_*y*_TiS_3 − *x*_Se_*x*_. **a** Projected density of states (pDOS) of Li_2_TiS_3_, Li_2_TiSe_3_, and Li_2_TiS_1.5_Se_1.5_. **b** Fukui functions computed to probe the electronic states involved in the oxidation process for the Li_2_TiS_3_, Li_2_TiSe_3_, and mixed Li_2_TiS_1.5_Se_1.5_ electrodes: these results clearly show that the holes generated upon oxidation (yellow volume) are mainly localized on the Ch (S and Se) anions for the pure-S and pure-Se electrodes, while the transition metal (Ti) is also involved for the mixed S/Se phases. Note that the Se selectivity in the mixed S/Se electrode is also clearly seen with a larger hole density around Se (pink atoms) much larger than around S (green atoms), therefore highlighting the Se selectivity in the mixed phases. **c** Atomic Bader net populations of the most oxidized S and Se atoms in the structure as a function of the Li content showing that Se is much more oxidized and S is much less oxidized in mixed S/Se compared to pure-S and pure-Se phases for an equivalent number of extracted lithium. All calculations were performed with the metaGGA SCAN functional (see “Methods” section).

But still, the electrochemical bell-shaped activation in Li_2_TiS_3 − *x*_Se_*x*_ needs to be investigated by studying the underlying mechanisms. Spin-polarized DFT calculations were performed on the layered Li_*y*_TiS_3 − *x*_Se_*x*_ phases (*y* = 0.5, 1, 1.5, 2; *x* = 0–3; 0.25)) using the VASP program package (see Supplementary information for details)^34–36^. Having first ensured that the unit-cell parameters obtained from full structural relaxation match the experimental data, we computed the projected density of states (Fig. 7a). Surprisingly, all pristine Li_2_TiS_3 − *x*_Se_*x*_ compounds show very similar semimetallic or small gap semiconducting electronic ground states with the occupied *Ch*^2−^ *p* and the empty Ti^4+^ 3*d* bands lying below and above the Fermi level, respectively. While the bandgap is slightly larger for the Li_2_TiS_3_ end-member as compared to the Li_2_TiS_1.5_Se_1.5_, such differences cannot explain the different electrochemical activity of these phases. The Fukui functions were thus computed for the Li_2_TiS_3 − *x*_Se_*x*_ series to probe the nature of the electronic states involved in the oxidation process. Fukui functions measure the variation of a system electron density upon charge variation (hole or electron addition) and are therefore typical electrochemical descriptors that allow identifying the redox centers of an electrochemical reaction. Computed for the two Li_2_TiS_3_ and Li_2_TiSe_3_ end-members, they reveal that S and Se are the redox centers of the oxidation process, as holes are mainly localized on the *Ch p* lone-pair states (see Fig. 7b). Their nonbonding character (unhybridized with the metal 3*d* orbitals), in the absence of an electron mediator (d^0^ metal), is consistent with the near absence of electrochemical activity for the pure-S and Se end-members.

Strikingly, such a scenario drastically changes when S is partially substituted by Se, which results in locally distorted Ti environments (reduction in symmetry) due to the formation of heteroleptic Ti*Ch*_6_ octahedra. The Fukui function computed for the mixed Li_2_TiS_1.5_Se_1.5_ phases now shows the participation of both the titanium and the chalcogens in the early stage of oxidation (see Fig. 7b). The nature of the electronic states around the Fermi level is changed from localized lone-pair states in pure-S and Se phases to hybridized Ti(*3d*)/Se(4*p*)/S(3*p*) in the mixed phases, which is fully consistent with the evolution of the *g*-factor deduced from EPR measurements. Hence, despite the apparent similarity of the density of states computed for the whole Li_2_TiS_3 − *x*_Se_*x*_ series, substantial differences occur in the nature of the electronic states involved in the oxidation process through an internal redox process that is activated by the symmetry lowering induced by the heteroleptic Ti*Ch*_6_ environments. This explains the activation of the electrochemical activity of the S/Se mixed electrodes. The internal redox in pristine Li_2_TiS_3 − *x*_Se_*x*_ is corroborated by Bader atomic charge analysis performed on the whole Li_2_TiS_3 − *x*_Se_*x*_ series as a function of *x* (Supplementary Figure 22), which confirms the evolution of the oxidized Se^*n−*^ species deduced from XPS/HAXPES measurements.

Lastly, the anionic band of the mixed S/Se electrodes is mainly constituted of S 3*p* states in its lower energy part and Se 4*p* states in its higher energy part, due to the different electronegativity of the two chalcogen atoms (Fig. 7a). Consequently, the Se subnetwork is selectively oxidized compared to the S subnetwork (Fig. 7c). Further investigation of the delithiation mechanism in these intriguing materials requires a thorough investigation by itself of both the statistical disorder of S/Se and the Li/Ti migration within the metallic layer and will be reported in an incoming publication.

Altogether, these computational results allow rationalizing the observed bell-shaped variation of the electrochemical activity of the electrodes. According to our results, anionic oxidation should be prevented in regular TiS_6_ or TiSe_6_ environments (hole localized in nonbonding *p* states) while it is activated preferentially on Se in distorted TiS_6 − *n*_Se_*n*_ environments (hole delocalization in hybridized Ti(*d*)/Se(*p*) states). Keeping in mind that the statistical occurrence of the distorted configurations increases with the Se content up to *x* = 1.5 and then decreases from *x* = 1.5 to 3, this explanation is consistent with the electrochemical activity of the Li_2_TiS_3 − *x*_Se_*x*_ phases following the bell-shaped behavior observed experimentally. Finally, the obvious evidence of having two coexisting ligand activities within the same compounds comforts previous theoretical models of Li-rich oxides that rely on nonuniform holes repartitions (i.e., coexisting domains of oxidized (O_2_^*n*−^) and nonoxidized (O_2−_) oxygen as opposed to uniformly oxidized (O_2_)^*n*−^ oxygen).

In all, these results demonstrate that S/Se substitution in Li_2_TiS_3_ can overcome an unfavorable band positioning to eventually trigger electrochemical activity. With the aim of checking the robustness of this novel strategy to stimulate anionic redox activity, we continued by investigating the Zr and Hf homologs. Compared to titanium, Zr and Hf are more electropositive and show correspondingly *d* bands positioned at higher energy, hence raising the question of how Se substitution would reshuffle the relative band positions. Here, we synthesized the Li_2_*M*S_3 − *x*_Se_*x*_ (*M* = Zr, Hf; *x* = 0, 1, 2, 3) series by solid-state reaction (see Supplementary information). Both sulfides, Li_2_ZrS_3_ and Li_2_HfS_3_, were briefly mentioned before, but in neither case structural or electrochemical characterization was attempted^37,38^. As in the case of Li_2_TiS_3 − *x*_Se_*x*_, full solid solutions form between the sulfide and selenide end-members (Supplementary Figure 23a–d). The end-members crystallize in layered O3-type structures alike Li_2_TiS_3_, which we refined in the $R\bar{3}m$ space group from SXRD data, not taking into account cation ordering (Supplementary Figures 24–27 and Supplementary Tables 11–14). The electrochemical activities of Li_2_ZrS_3_ and Li_2_HfS_3_ are negligible and barely increase upon Se substitution (Supplementary Figure 23e–f). Interestingly, a change of band positioning is manifested in a change of color from gray-black (Li_2_TiS_3_) to yellow-off-white (Li_2_ZrS_3_) to red-greyish (Li_2_HfS_3_). As Zr and Hf are more electropositive than Ti, the conduction band, composed of the 4d^0^ band (Li_2_ZrS_3_) or the 5d^0^ band (Li_2_HfS_3_), is positioned higher in energy compared to that of Li_2_TiS_3_ (Supplementary Figure 28). This greater bandgap cannot be sufficiently mitigated by Se substitution, rendering all materials inactive.

We have shown the feasibility to synthesize the full Li-rich layered chalcogenide Li_2_TiS_3 − *x*_Se_*x*_ series and found that the partial substitution of S by Se triggers the electrochemical activity of Li_2_TiS_3_ with a bell-shaped electrochemical activity. Li_2_TiS_2.4_Se_0.6_ can deliver a gravimetric capacity of up to 260 mAh/g that compares to what has been achieved for layered oxides but without the voltage fade and high polarization penalties. It is shown, via combined XPS/HAXPES, EPR and NMR techniques, that this capacity originates from a complex balance between anionic (S^2−^/S^*n*−^; Se^2−^/Se^*n−*^, *n* < 2) and cationic redox (Ti^3+/4+^) redox processes. We propose anion substitution as a new strategy to unlock reversible anionic redox in Li-rich materials by changing not only the energy but also the symmetry of the electronic band. This triggers the dynamic involvement of the electronic TM states, without which anionic redox cannot happen. As importantly, our low-temperature EPR/NMR measurements provide experimental evidence of internal ligand to metal charge transfer in this highly covalent system through temperature-driven electron localization, which indicates that band alignment and positioning is a dynamic process escaping simple description and assignment of oxidation states. These compelling findings offer a fertile playground for shedding light on the fundamentals of anionic redox. Performance-wise, both LTFS and Li_2_TiS_3 − *x*_Se_*x*_ show comparable sustainable specific energy density. So in short, this type of chalcogenide chemistry opens the door to solid-state electrochemists for tuning cationic/anionic band positions (and the redox potentials) and hence increasing the electrochemical capacities of Li-rich sulfides. Chemical compatibility with S-based ionic conductors in solid-state batteries could be a surplus, rekindling the interest within the battery community.

## Supplementary information

Supplementary Information

## Data Availability

The authors declare that the main data supporting the findings of this study are available within the article and its Supplementary information. Extra data are available from the corresponding authors on reasonable request.
