# Supplementary material for: Activation of anionic redox in d0 transition metal chalcogenides by anion doping
Source: Nat Commun. 2021 Sep 16;12:5485. doi: 10.1038/s41467-021-25760-8 (PMC8445930; doi:10.1038/s41467-021-25760-8)
Supplement: Supplementary file 1 — Supplementary Information [file 41467_2021_25760_MOESM1_ESM.pdf]

Supplementary information

Activation of anionic redox in d^0 transition metal chalcogenides by anion doping

Bernhard T. Leube[1,2], Clara Robert[2,3], Dominique Foix[2,4], Benjamin Porcheron[2,5], Remi Dedryvère[2,4], Gwenaëlle Rousse[1,2,6], Elodie Salager,[2,5] Pierre-Etienne Calbenguen[7], Artem M. Abakumov[8], Hervé Vezin[9], Marie-Liesse Doublet[2,3], and Jean-Marie Tarascon[1,2*]

[1] Collège de France, Chaire de Chimie du Solide et de l'Energie, UMR 8260, 11 Place Marcelin Berthelot, 75231 CEDEX 05 Paris, France
[2] Réseau sur le Stockage Electrochimique de l'Energie (RS2E), FR CNRS 3459, 33 Rue Saint Leu, 80039 Amiens, France
[3] ICGM, Univ Montpellier, CNRS, ENSCM, Montpellier, France
[4] IPREM/ECP (UMR 5254), Université de Pau, 2 Avenue Pierre Angot, 64053 Pau Cedex 9, France
[5] CNRS-CEMHTI, Université d'Orléans, Orléans, France
[6] Sorbonne Université, 4 Place Jussieu, F-75005 Paris, France
[7] Umicore, New Business Incubation, 31 rue Marais, 1000 Brussels, Belgium
[8] Center for Energy Science and Technology, Skolkovo Institute of Science and Technology, Nobel str. 3, 121205 Moscow, Russia
[9] Université Lille Nord de France, CNRS UMR8516, LASIRE, Villeneuve d'Ascq, France

[*] e-mail : jean-marie.tarascon@college-de-france.fr

Methods.

General procedure. All reagents were used as received from the vendors and all sample manipulations were carried out in glove boxes. In general, batches of typically 500 - 1000 mg were prepared by mixing and grinding (in case of powders) the reagents in appropriate stoichiometric ratios. The reactions mixtures were then placed in alumina crucibles as powders, sealed in evacuated quartz tubes under vacuum ($p < 10^{-4}$ mbar) and heated to the indicated temperature.

Li_2S. Lithium sulfide was synthesized by heating lithium carbonate (Li_2CO_3, Sigma-Aldrich, > 99%) under a stream of argon/CS_2 (Sigma-Aldrich) vapor at 923 K for 5 h.

Li_2Se. Lithium selenide was synthesized by heating lithium ribbon (Li, Sigma-Aldrich, 99.9%) and selenium powder (Se, Alfa Aesar, 99.999%) at 673 K for 12 h.

$TiSe_2$. Titanium selenide was synthesized by heating titanium (Ti, Sigma-Aldrich, 99. 98%) and selenium powder (Se, Alfa Aesar, 99.999%) in an evacuated quartz tube at 673 K for 12 h.

Zr*Ch*$_2$ (*Ch*= S, Se). Zirconium chalcogenides were synthesized by heating zirconium wire (Zr, Alfa Aesar, 99.2%) with sulphur (S, Sigma-Aldrich, 99.98%) or selenium powder at 1273 K for 12 h applying a very low heating rate of 0.5 K/min.

Hf*Ch*$_2$ (*CH*= S, Se). Hafnium chalcogenides were synthesized by heating hafnium powder (Hf, Alfa Aesar, 99.6%) and sulphur or selenium powder at 1273 K for 12 h.

$Li_2MS_{3-x}Se_x$ ($0 < x < 3$, *M*= Ti, Zr, Hf). Hand ground mixtures of in-house prepared Li_2S, Li_2Se, $TiSe_2$, ZrS_2, $ZrSe_2$, HfS_2, $HfSe_2$ and commercially available TiS_2 (Sigma-Aldrich, 99.9%) were heated to 973 K for 48 h (heating rate: 5K /min). While usually a cooling rate of 5 K/min was applied, quenching the samples from 973 K in a water bath did not lead to perceptible differences in the PXRD patterns. Only Li_2TiSe_3 was fired at a reduced temperature of 873 K for 72 h to reduce the formation of impurity phases.

Chemical delithiation of $Li_2TiS_{2.4}Se_{0.6}$. To chemically synthesize $Li_{0.3}TiS_{2.4}Se_{0.6}$ delithiation reactions were performed by stirring $Li_2TiS_{2.4}Se_{0.6}$ (*M*= 186.8 g/mol, *m*= 1200 mg, *n*= 6.424 mmol, 1.00 eq.) and I_2 (Alfa Aesar, *M*= 253.8 g/mol, *m*= 1650 mg, *n*= 6.5 mmol, 1.01 eq) in 10 mL Acetonitrile at RT for 24 h. The centrifuged material was washed with Acetonitril (3x 10 mL) and dried for 2 h under vacuum.

Chemical re-lithiation of $Li_{0.3}TiS_{2.4}Se_{0.6}$. To chemically obtain re-lithiated $Li_2TiS_{2.4}Se_{0.6}$, chemically delithiated $Li_{0.3}TiS_{2.4}Se_{0.6}$ (*M*= 172.2 g/mol, *m*= 25 mg, *n*= 0.145 mmol, 1.00 eq.) was suspended in 1 mL dry hexane. *n*-Butyllithium (Sigma-Aldrich, *c*= 1.6 mol/L in hexane, *V*= 0.23 mL, *n*= 0.363 mmol, 2.50 eq.) was added slowly, stirred at RT for 8 h and finally washed with hexane (3x 1 mL). To equilibrate the internal lithium concentration differences, the material was pressed into a pellet and drowned in LP30 (5 drops). After 72 h the reaction product was washed with DMC (3x 1 mL) and dried for 2 h under vacuum.

Characterization.

X-ray powder diffraction. Synchrotron X-ray diffraction (SXRD) patterns were recorded at the 11-BM of the Advanced Photon Source (APS), Argonne National Laboratory. The samples were sealed in quartz capillaries (r_{cap}= 0.25 mm) and measured in transmission mode (λ= 0.457889 Å). For in house powder X-ray diffraction (PXRD) experiments samples were enclosed in an airtight electrochemical cell with a Be window. The data was collected in reflection mode in Bragg-Brentano geometry using a Bruker D8 Advance diffractometer equipped with a Cu-K_{α} source (λ_1= 1.54056 Å, λ_2= 1.54439 Å) and a LynxEye detector. Analysis and refinements of the as collected diffraction patterns were carried out using the FullProf program suite.

Neutron powder diffraction. A constant wavelength neutron powder diffraction (NPD) pattern of approximately 1 g pristine $Li_2TiS_{2.4}Se_{0.6}$ was recorded on the D2B diffractometer (λ= 1.594384 Å), Institut Laue-Langevin.

Electrochemical characterization. All galvanostatic electrochemical cycling of $Li_2TiS_{3-x}Se_x$ was performed with BioLogic potentiostats in Li half-cell configuration. Prior cell assembly the as synthesized cathode materials were thoroughly ground with 15 wt% electrically conducting carbon Super-P. Typically Swagelok cell type cells with loadings of 5-10 mg of the cathode material, Whatman GF/D borosilicate glass fiber membranes as the separator, LP30 (Elyte, 1 M $LiPF_6$ in 1/1 wt/wt DMC/EC, 18 drops ≈ 0.2 mL) as electrolyte and a disc of metallic Li as anode were assembled in an Ar-filled glovebox. The cells were allowed to rest for 30 min and were then cycled between 3.0 V and 1.7 V at a *C/*10 rate, corresponding to the removal of 1 Li per formula unit. *Operando* PXRD experiments were performed at a *C*/20 rate with a PXRD scan-time of 1 h, which corresponds to a change in Li composition of Δy= 0.05 Li for each collected pattern. For *ex-situ* experiments the cathode materials were recovered after cycling, washed with dimethyl carbonate (DMC, Sigma-Aldrich, >99%) and dried under vacuum. Galvanostatic intermittent titration technique (GITT) experiments were run on the second electrochemical cycle of the respective material with galvanostatic pulses at rates of *C/10* for 1.5 h followed by a 4 h open circuit step.

HAXPES/XPS. HAXPES measurements were carried out at the GALAXIES beamline of SOLEIL synchrotron, France. Photon excitation energy of hυ = 10.0 keV was obtained from the third-order reflection of Si(111) double-crystal monochromator. Photoelectrons were analyzed by a SCIENTA EW4000 spectrometer, with an energy resolution of 0.22 eV from the Au Fermi edge. No charge neutralizer was required, and the analysis chamber pressure was maintained around 10^{-8} mbar during the measurements. Home XPS measurements were carried out with a THERMO Escalab spectrometer, using focused monochromatic Al Kα radiation (hν = 1486.6 eV). Peaks were recorded with constant pass energy of 20 eV. The pressure in the analysis chamber was around 5×10^{-8} mbar. The binding energy scale was calibrated using the S $2p_{3/2}$ component of the sulfides S^{2-} (at 161.1 eV) in the material. The spectra were fitted using a minimum number of components. For both HAXPES/Home XPS experiments, several spectra were recorded at different times to check that the samples were not subject to degradation during the X-ray irradiation.

EPR spectroscopy. EPR experiments were performed using a Bruker ELEXYS E580 spectrometer operating at 9.7 GHz. CW spectra were recorded at room temperature and 110K CW and were acquired with microwave power of 2mW and an amplitude modulation of 2G . The 2-pulses echo field sweep experiments were respectively measured with standard Hahn echo experiments with respectively $\pi/2$ and π pulses of 16 and 32 ns and a τ value of 200 ns. These spectra were collected at 5K using a ColdEdge cryofree cryostat.

DFT. Spin-polarized density functional theory (DFT) calculations as implemented in VASP (Vienna ab initio simulation package)[1,2] were performed, using the projected augmented wave method (PAW).[3] Different XC functionals were used to check the importance of electron correlation in the systems: the generalized gradient approximation of Perdew−Burke−Ernzerhof (PBE)[4] in conjunction with the rotationally invariant Dudarev method (DFT+U)[5] and the metaGGA (SCAN)[6] functional. As a consequence of the relatively strong covalence of the Ti-S and Ti-Se bonds, the self-interaction error is limited in these systems thanks to the efficient screened of the on-site coulombic repulsions by the long-range electron delocalization over the structure. As a consequence, DFT+U with moderate U_{eff}(Ti) = 2eV and SCAN lead to similar results, as depicted below on the atom-projected Density of States (pDOS) computed with the two functionals. Note also that negligible effect of spin-orbit coupling was predicted by our calculations on the electronic structure of Se-based electrodes.

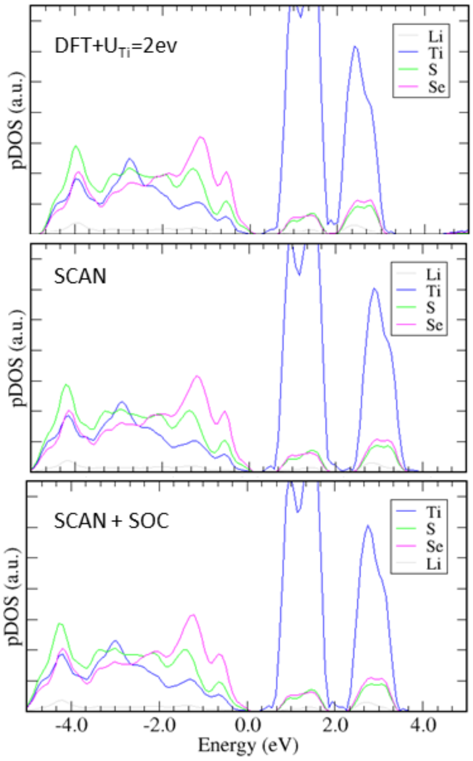

The layered $Li_{2-y}TiS_{3-x}Se_x$ phases with x varying from 0 to 3 (step 0.25) where built with different S/Se ordering. All input structures were relaxed until the forces on atoms are smaller than 3.10^{-3} eV/Å. The cut-off value for energy was set to 600 eV and the k-point mesh for Brillouin zone integration was converged to a grid density of at least 1000/at. Fukui functions were computed on the pristine $Li_2TiS_{3-x}Se_x$ phases in order to identify the redox centre involved in the first step of oxidation.

TEM. TEM measurements were performed on pristine $Li_2TiS_{2.4}Se_{0.6}$, charged $Li_{0.3}TiS_{2.4}Se_{0.6}$ (after 40 electrochemical cycles) and discharged $Li_2TiS_{2.4}Se_{0.6}$ (after 40 electrochemical cycles). The samples were prepared by dispersing the powder in DMC (dimethyl carbonate) in an agate mortar and depositing drops of this suspension onto a carbon film supported by a copper grid. The samples were transferred from the Ar-filled glove box to the microscope column using a Gata vacuum transfer holder completely excluding contact with air. Electron diffraction (ED) patterns, high-angle annular dark-field scanning transmission electron microscopy (HAADF-STEM) images and scanning transmission electron microscopy (STEM-EDX) compositional maps were acquired on a probe aberration-corrected FEI Titan

Themis Z electron microscope operated at 200 kV and equipped with a Super-X system for energy-dispersive X-ray (EDX) analysis.

NMR. The NMR spectra of Li_2TiSe_3 were recorded on a Bruker 4.7 T (200 MHz) Avance III spectrometer operating at 77.79 MHz for ^{7}Li and 38.22 MHz for ^{77}Se. The Li_2TiSe_3 pristine powder was packed in a 1.3mm and a 4mm (outer diameter) zirconia rotor in an Argon glovebox. The ^{77}Se MAS-NMR spectrum was recorded using a Bruker 4mm HX probe spinning under nitrogen gas at 12.5 kHz MAS. The ^{77}Se spins were saturated, then a delay of 25 s allowed for reasonable relaxation of the spins (not quantitative), and finally a Hahn-echo sequence synchronized with one rotor period (total evolution time: 160 us) was used, with a RF pulse of 4.56 us (55 kHz RF power). 1424 transients were added and processed with an exponential apodization (100 Hz). The spectrum was referenced using H_2SeO_3 at 1288 ppm. The ^{7}Li MAS-NMR spectrum was recorded using a Bruker 1.3mm HX probe spinning under nitrogen gas at 62.5 kHz. A single RF pulse of duration 1.85 us (strength 135 kHz) was applied to record the spectrum; 8 transients were co-added. The ^{7}Li spins were saturated before each transient, followed by a recovery of 25 s - sufficient for full relaxation of the ^{7}Li spins. The spectrum was referenced using a 1 $mol.L^{-1}$ aqueous solution of LiCl at 0 ppm. The ^{7}Li static NMR spectra and longitudinal relaxation measurements were performed with a Bruker single channel probe, using a 5mm horizontal solenoid coil. The 4mm rotor was introduced in a 5mm (outer diameter) glass tube, sealed with a cap and parafilm. A saturation recovery experiment was performed, with an excitation RF pulse of 7.125 us (RF power: 35 kHz). The sample was under N_2 flux over the whole experiment, refrigerated using a Bruker BCU I fridge and a N_2 heat exchanger. At each temperature the sample was left to thermally equilibrate during at least 10 minutes. Temperature was calibrated using the ^{207}Pb NMR spectrum of $Pb(NO_3)_2$ measured in similar conditions. The recovery was fitted in the Topspin software with 2 components. The spectra were fitted using the dmfit software.[7]

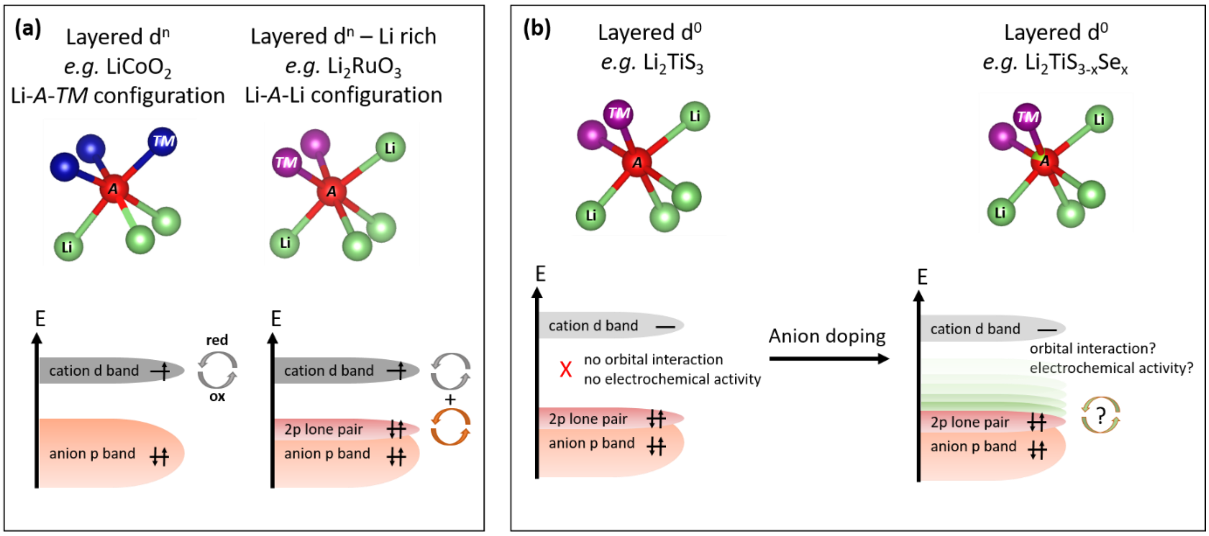

Supplementary Figure 1: Schematic representation of origin of anionic redox in conventional lithium rich metal oxides (a) by going from stoichiometric layered transition metal oxides (cationic redox) to lithium rich transition metal oxides, the local coordination and therefore the bonding of the anion A is altered. 2p lone pairs, which were previously involved in bonding to the d orbitals of the transition metal TM, are in consequence liberated on the anion. These 2p lone pairs sit on top of the anion sp band and can act as electron reservoirs and thus potentially participate in electrochemical activity (cationic + anionic redox). (b) Proposed strategy of band alignment by anion doping to induce electrochemical activity in d^0 metal lithium rich chalcogenides.

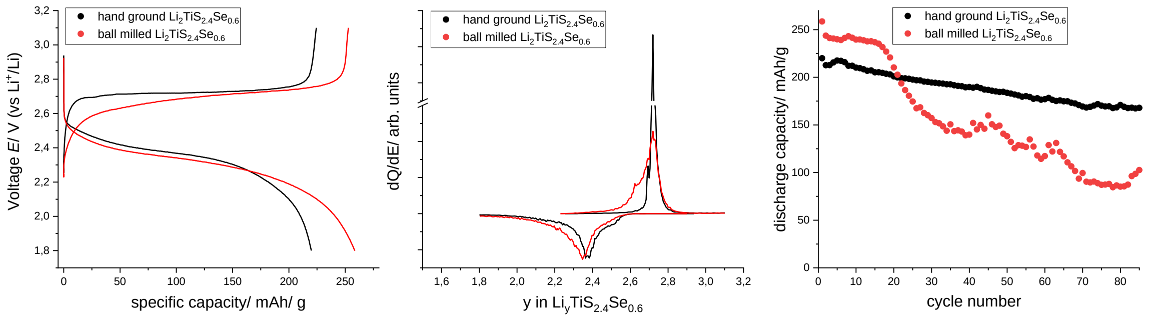

Supplementary Figure 2: Comparison of hand ground and ball milled cycling of $Li_2TiS_{2.4}Se_{0.6}$: in both cases $Li_2TiS_{2.4}Se_{0.6}$ was mixed with 15 wt% Carbon Super-P and then hand ground for ten minutes or ball milled for 30 minutes respectively (a) voltage profile of first electrochemical cycle (b) differential capacity during first cycle (c) long-term cyclability.

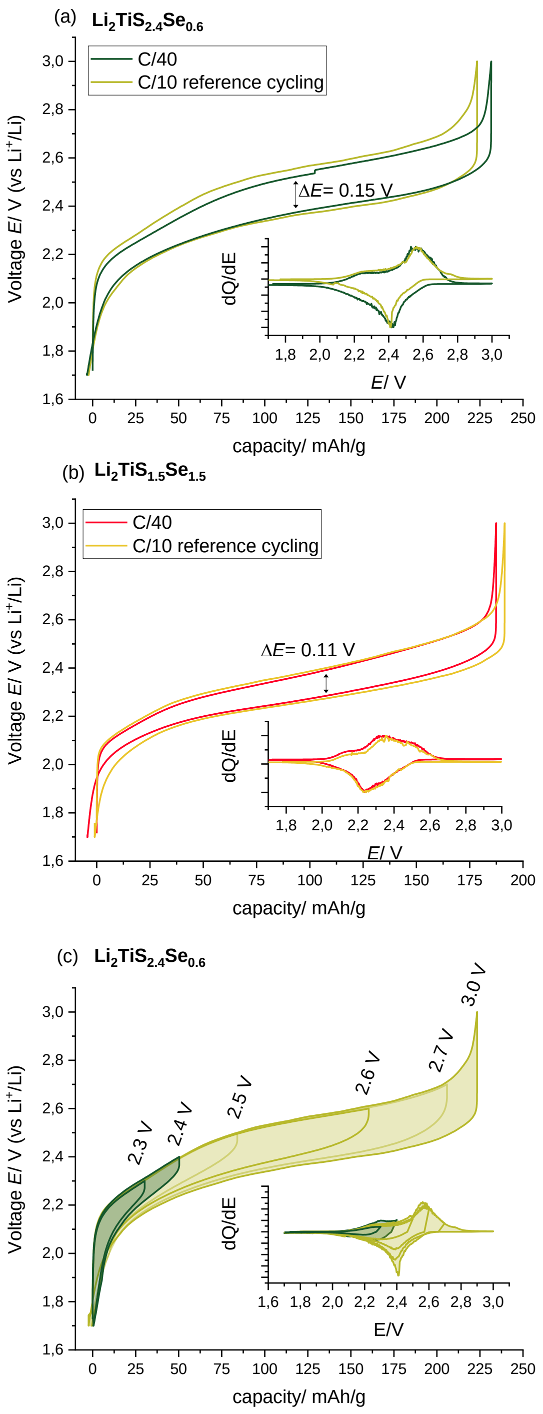

Supplementary Figure 3: expanded electrochemical characterization of $Li_2TiS_{3-x}Se_x$ (a) low C/40 rate cycling of $Li_2TiS_{2.4}Se_{0.6}$. (b) low C/40 rate cycling of $Li_2TiS_{1.5}Se_{.15}$. (c) voltage opening experiment on $Li_2TiS_{2.4}Se_{0.6}$.

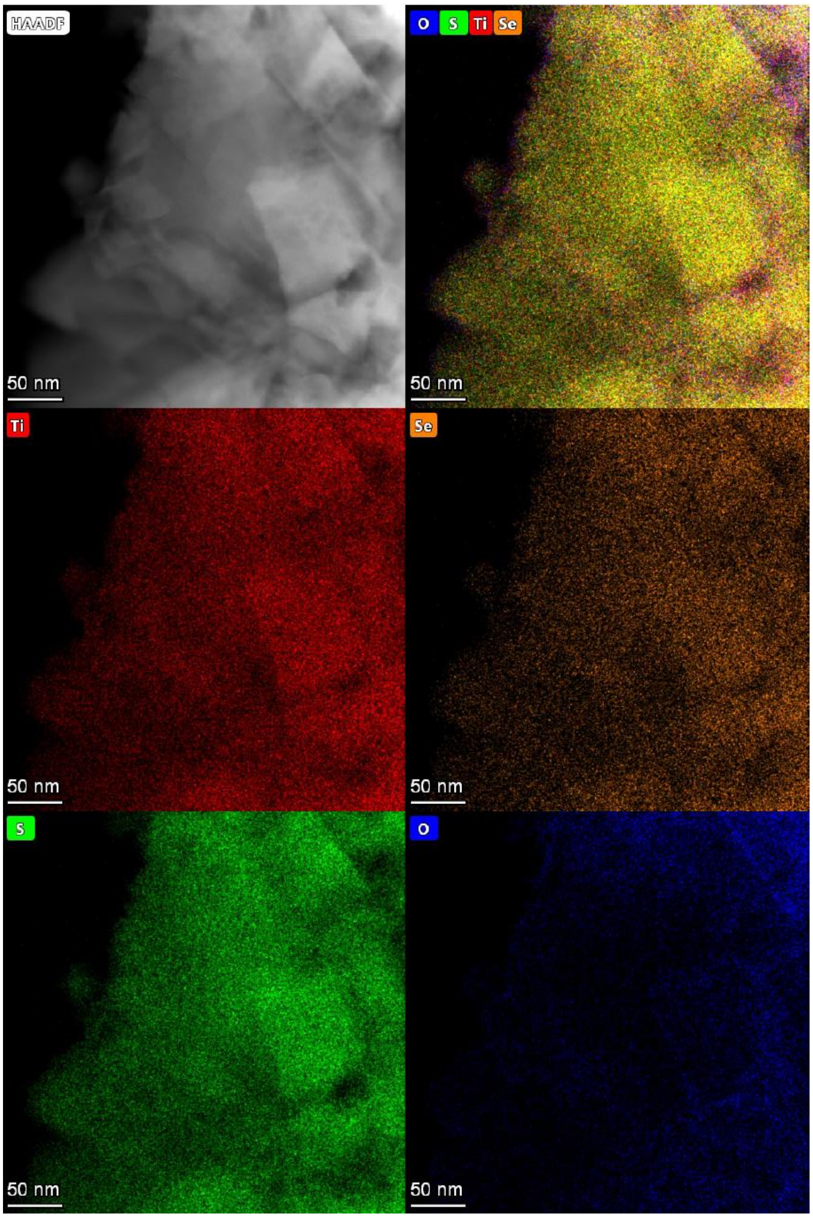

Supplementary Figure 4: HAADF-STEM image of pristine $Li_2TiS_{2.4}Se_{0.6}$, the corresponding EDX elemental maps for Ti, S, Se and O, and the mixed color-coded compositional map.

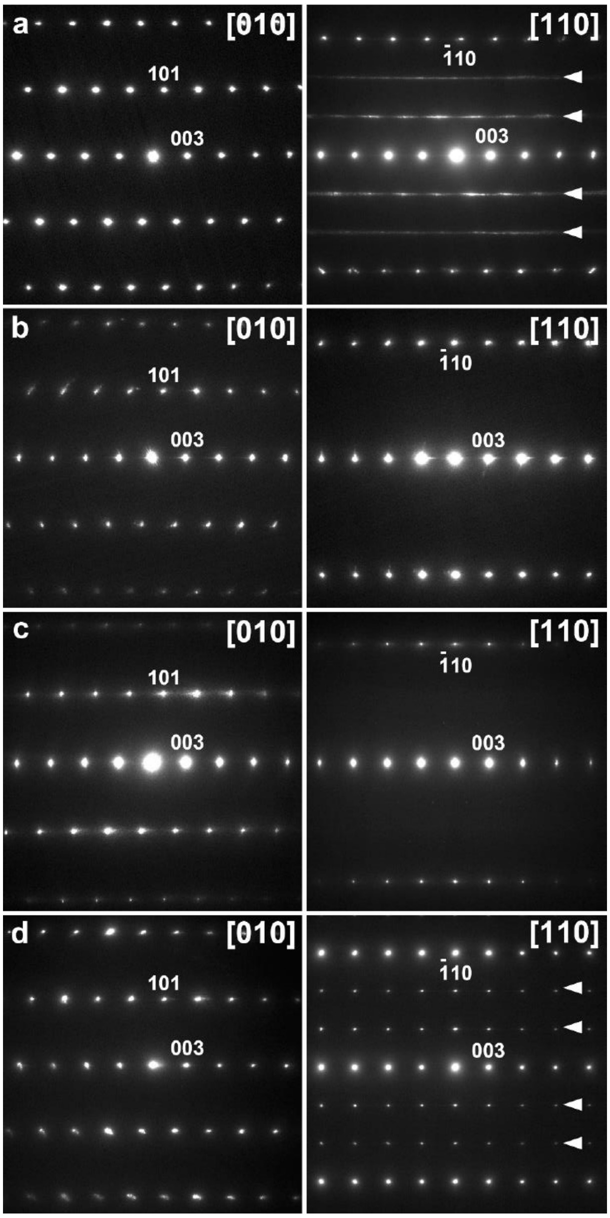

Supplementary Figure 5: ED (electron diffraction) patterns of $Li_yTi_{2.4}Se_{0.6}$. (a) pristine (b) charged after 40 cycles (c) chemically delithiated and (d) discharged after 40 cycles indexed with the $R\bar{3}m$ space group corresponding to an O3 type layered structure. The arrowheads mark diffuse intensity associated with honeycomb Li-Ti ordering in the $[Li_{1/3}Ti_{2/3}]Ch_2$ slabs.

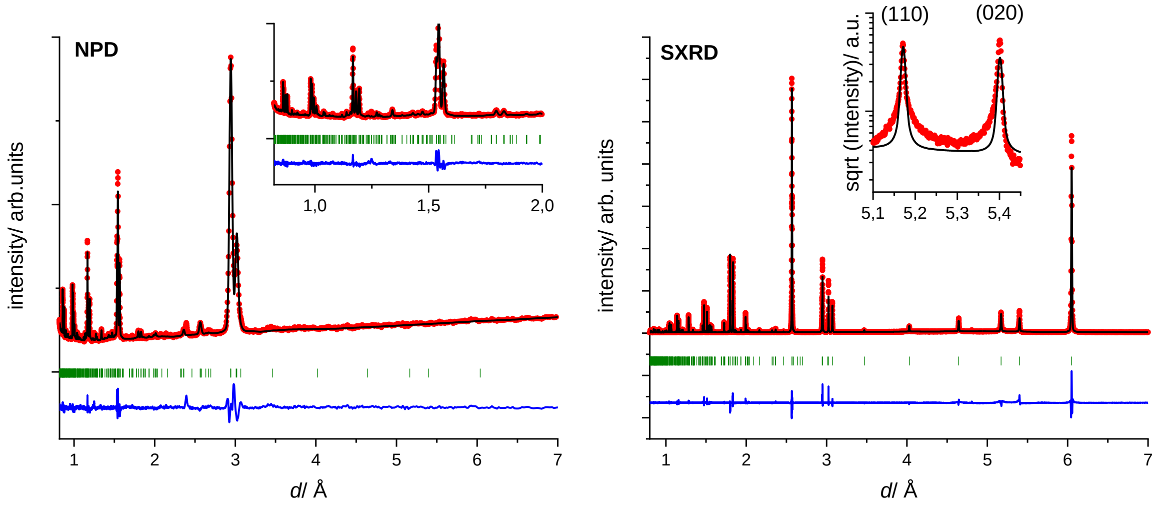

Supplementary Figure 6: Combined Rietveld refinement of $Li_2TiS_{2.4}Se_{0.6}$. (a) NPD fit (b) SXRD fit. The insert in (b) shows superstructure reflections with significant peak broadening due to stacking faults.

Supplementary Table 1: Crystallographic data of $Li_2TiS_{2.4}Se_{0.6}$ as obtained from a combined NPD and SXRD Rietveld refinement at 300 K in C2/m (a= 6.22537(19) Å, b= 10.7876(3) Å, c= 6.38994(13) Å, β= 109.0949(18)°, χ^2(SXRD)= 3.63, χ^2(NPD)= 3.77).

Atom	Wyckoff site	x	y	z	occupancy	U_{iso}/ Å^2
S1	4*i*	0.2314(2)	0.0	0.23560(18)	0.8076(19)	0.008(3)
Se1	4*i*	0.2314(2)	0.0	0.23560(18)	0.1924(19)	0.008(3)
S2	8*j*	0.25301(14)	0.32575(8)	0.23484(11)	0.7962(10)	0.0129(2)
Se2	8*j*	0.25301(14)	0.32575(8)	0.23484(11)	0.2038(10)	0.0129(2)
Ti1	4*g*	0.0	0.16815(14)	0.0	0.8685(13)	0.16815(14)
Li1	4*g*	0.0	0.16815(14)	0.0	0.1315(13)	0.16815(14)
Ti2	2*b*	0.0	0.5	0.0	0.263(3)	0.0237(9)
Li2	2*b*	0.0	0.5	0.0	0.737(3)	0.0237(9)
Li3	2*c*	0.0	0.0	0.5	1.0	0.0237(9)
Li4	4*h*	0	0.3288(11)	0.5	1.0	0.0237(9)

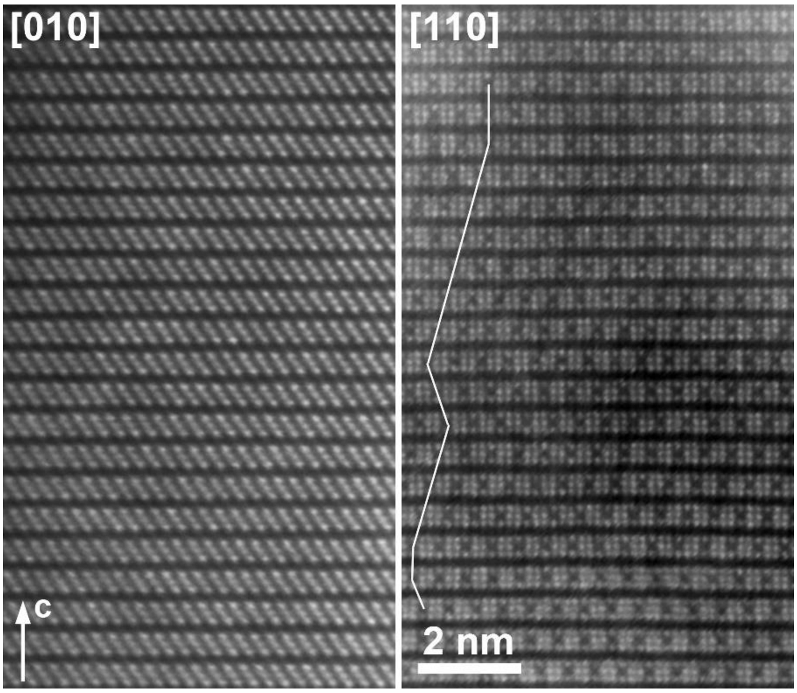

Supplementary Figure 7: [010] and [110] HAADF-STEM images of pristine $Li_2TiS_{2.4}Se_{0.6}$. The O3 structure is visible in the [010] image by lateral displacement of every successive layer by 1/3 of the interdot distance. Stacking faults in the honeycomb Li-Ti ordering are seen in the [110] image by random lateral displacements of the $[Li_{1/3}Ti_{2/3}]Ch_2$ blocks traced with the zig-zag line.

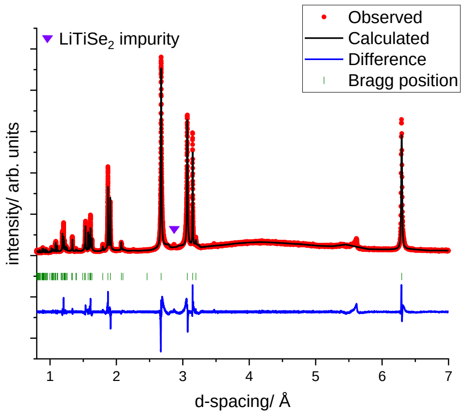

Supplementary Figure 8: Rietveld refinement of Li_2TiSe_3 in the $R\overline{3}m$ space group.

Supplementary Table 2: Crystallographic data of the Rietveld refinement of Li_2TiSe_3 in the $R\overline{3}m$ space group (a= 3.745179(15) Å, c= 18.88454(11) Å, χ^2(SXRD)= 4.24).

Atom	Wyckoff site	x	y	z	occupancy	U_{iso}/ Å^2
Se1	6*c*	0.0	0.0	0.25558(3)	1.0	0.00485(8)
Ti1	3*a*	0.0	0.0	0.0	0.66667	0.0175(4)
Li1	3*a*	0.0	0.0	0.0	0.33333	0.0175(4)
Li2	3*b*	0.0	0.0	0.5	1.0	0.01267

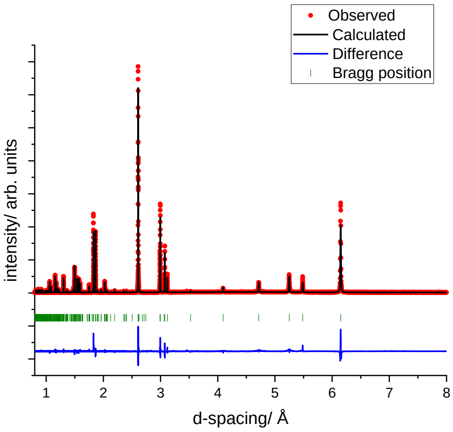

Supplementary Figure 9: Rietveld refinement of $Li_2TiS_{1.5}Se_{1.5}$ in C2/m.

Supplementary Table 3: Crystallographic data of the Rietveld refinement of $Li_2TiS_{1.5}Se_{1.5}$ in the C2/m space group (a= 6.326182(5) Å, b= 10.970604(8) Å, c= 6.506271(4) Å, β= 109.07001(5)°, χ^2(SXRD)= 5.22).

Atom	Wyckoff site	x	y	z	occupancy	U_{iso}/ $Å^2$
S1	4*i*	0.23010(12)	0.0	0.23664(12)	0.5	0.00907
Se1	4*i*	0.23010(12)	0.0	0.23664(12)	0.5	0.00907
S2	8*j*	0.25341(10)	0.32598(5)	0.23616(8)	0.5	0.01374
Se2	8*j*	0.25341(10)	0.32598(5)	0.23616(8)	0.5	0.01374
Ti1	4*g*	0.0	0.16808(11)	0.0	0.9214(12)	0.01673
Li1	4*g*	0.0	0.16808(11)	0.0	0.0786(12)	0.01673
Ti2	2*b*	0.0	0.5	0.0	0.157(2)	0.02329
Li2	2*b*	0.0	0.5	0.0	0.843(2)	0.02329
Li3	2*c*	0.0	0.0	0.5	1.0	0.03025
Li4	4*h*	0.0	0.3341(10)	0.5	1.0	0.03407

Supplementary Table 4: Bond distances in pristine $Li_2TiS_{3-x}Se_x$.

	Li_2TiS_3	$Li_2TiS_{2.4}Se_{0.6}$	$Li_2TiS_{1.5}Se_{1.5}$	Li_2TiSe_3
**Average Ti-*Ch***	**2.5014**	**2.5348**	**2.583**	**2.6137(4)**
Ti1- *Ch*1 (2x)	2.4515	2.4923(13)	2.53468	X
Ti1- *Ch*2 (2x)	2.4364	2.4650(13)	2.51387	X
Ti1- *Ch*2 (2x)	2.4392	2.4759(10)	2.52376	X
Ti2- *Ch*1 (2x)	2.5586	2.5906(14)	2.64610	X
Ti2- *Ch*2 (4x)	2.5613	2.5924(8)	2.63979	x
**Average Li-*Ch***	**2.6051**	**2.6220**	**2.6629**	**2.7377(4)**
Li3- *Ch*1 (2x)	2.5338	2.5535(14)	2.58596	X
Li3- *Ch*2 (4x)	2.6369	2.6580(18)	2.69701	X
Li4- *Ch*1 (2x)	2.7379	2.688(9)	2.62604	X
Li4- *Ch*2 (2x)	2.4359	2.510(8)	2.66083	X
Li4- *Ch*2 (2x)	2.6489	2.6643(10)	2.71047	x

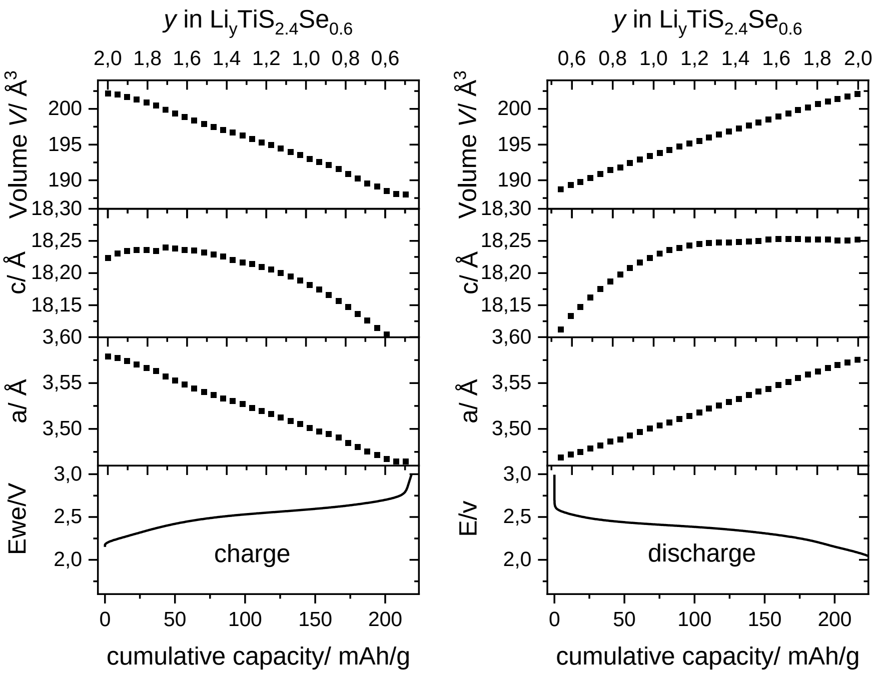

Supplementary Figure 10: Evolution of the lattice parameters of $Li_2TiS_{2.4}Se_{0.6}$ during the 2nd cycle of an operando PXRD experiment (space group: $R\overline{3}m$).

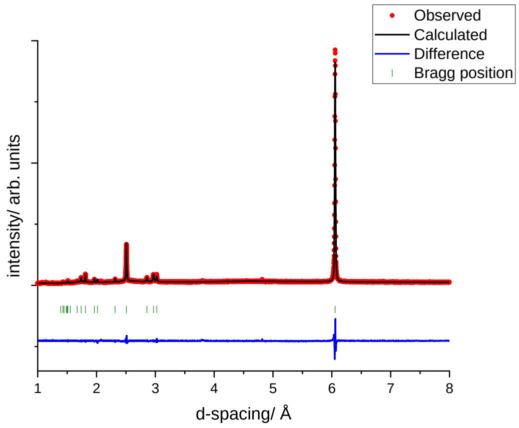

Supplementary Figure 11: LeBail refinement of chemically delithiated $Li_{0.3}TiS_{2.4}Se_{0.6}$ in the $R\overline{3}m$ space group (a= 3.4759(1) Å, c= 18.1388(5) Å).

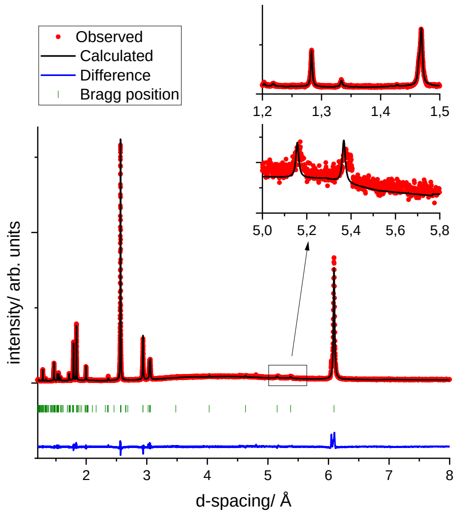

Supplementary Figure 12: Rietveld refinement of $Li_2TiS_{2.4}Se_{0.6}$ after one electrochemical cycle in C2/m.

Supplementary Table 5: Crystallographic data of the Rietveld refinement of discharged $Li_2TiS_{2.4}Se_{0.6}$ in the C2/m space group after one electrochemical cycle (a= 6.21022(2) Å, b= 10.73424(4) Å, c= 6.43228(2) Å, β= 108.7443(5)°, χ^2(SXRD)= 2.11).

Atom	Wyckoff site	x	y	z	occupancy	U_{iso}/ Å^2
S1	4*i*	0.2372(5)	0.0	0.2415(5)	0.8	0.00937
Se1	4*i*	0.2372(5)	0.0	0.2415(5)	0.2	0.00937
S2	8*j*	0.2477(4)	0.3292(2)	0.2316(3)	0.8	0.02679
Se2	8*j*	0.2477(4)	0.3292(2)	0.2316(3)	0.2	0.02679
Ti1	4*g*	0.0	0.1700(4)	0.0	0.729(2)	0.01751
Li1	4*g*	0.0	0.1700(4)	0.0	0.271(2)	0.01751
Ti2	2*b*	0.0	0.5	0.0	0.543(5)	0.01318
Li2	2*b*	0.0	0.5	0.0	0.457(5)	0.01318
Li3	2*c*	0.0	0.0	0.5	1.0	0.01956
Li4	4*h*	0.0	0.3149(16)	0.5	1.0	0.01956

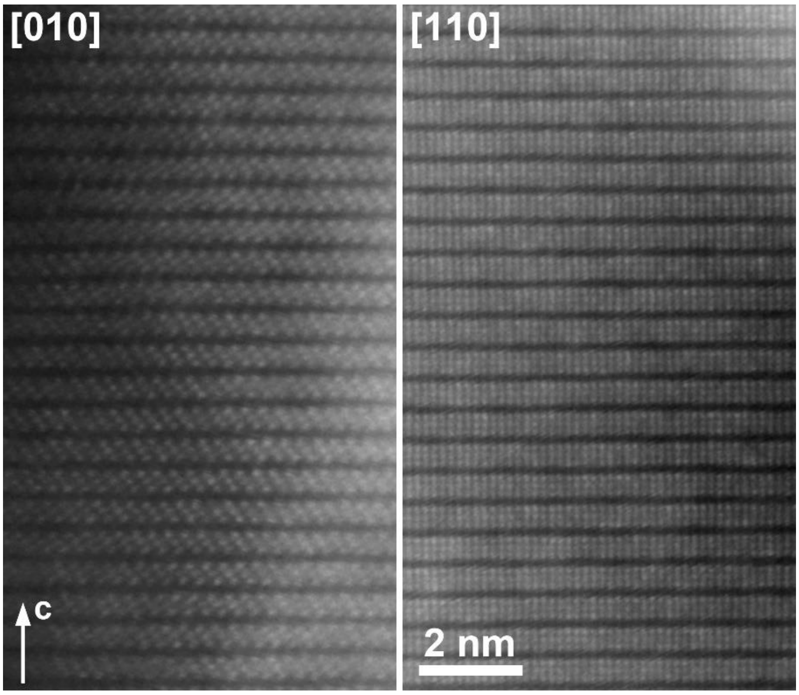

Supplementary Figure 13: [010] and [110] HAADF-STEM images of $Li_2TiS_{2.4}Se_{0.6}$, charged after 40 cycles. The O3-type stacking remains intact on charge as evidenced with the [010] image. Note that the characteristic “double dot” contrast along the $[Li_{1/3}Ti_{2/3}]Ch_2$ slabs in the [110] image is completely vanished indicating suppression of the Li-Ti honeycomb ordering (compare with *Supplementary Figure 7*).

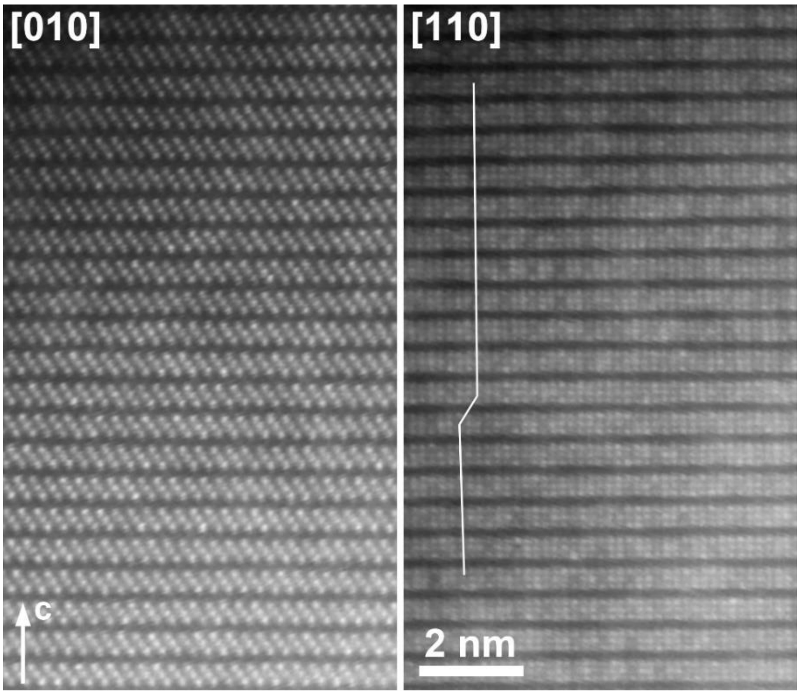

Supplementary Figure 14: [010] and [110] HAADF-STEM images of $Li_2TiS_{2.4}Se_{0.6}$, discharged after 40 cycles. Note that the characteristic “double dot” contrast along the $[Li_{1/3}Ti_{2/3}]Ch_2$ slabs is partially restored indicating restoration of the honeycomb Li-Ti ordering. Lateral shifts of the honeycomb layers due to stacking faults occur only occasionally as traced with the white line.

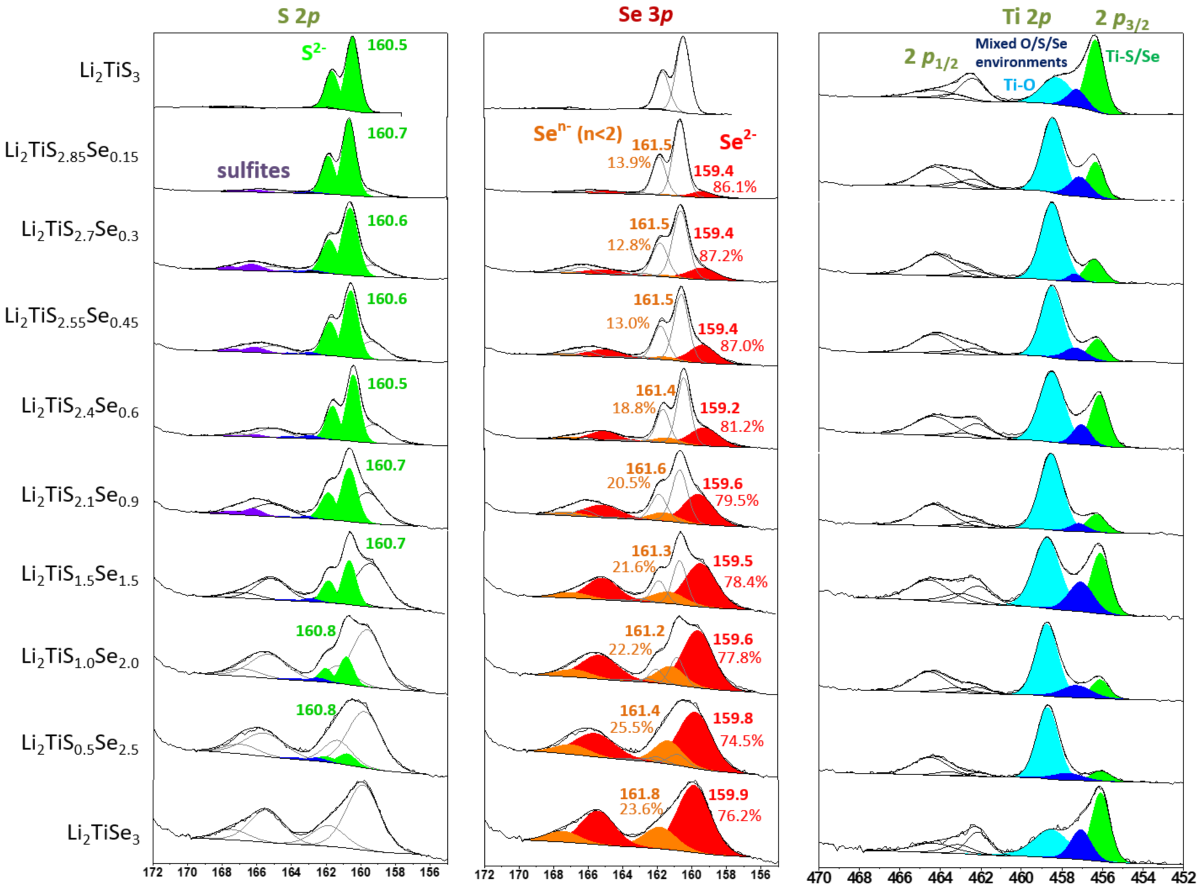

Supplementary Figure 15: XPS spectra of pristine materials in $Li_2TiS_{3-x}Se_x$. With increasing Se content an increasing amount of partially oxidized Se^{n-} is detected.

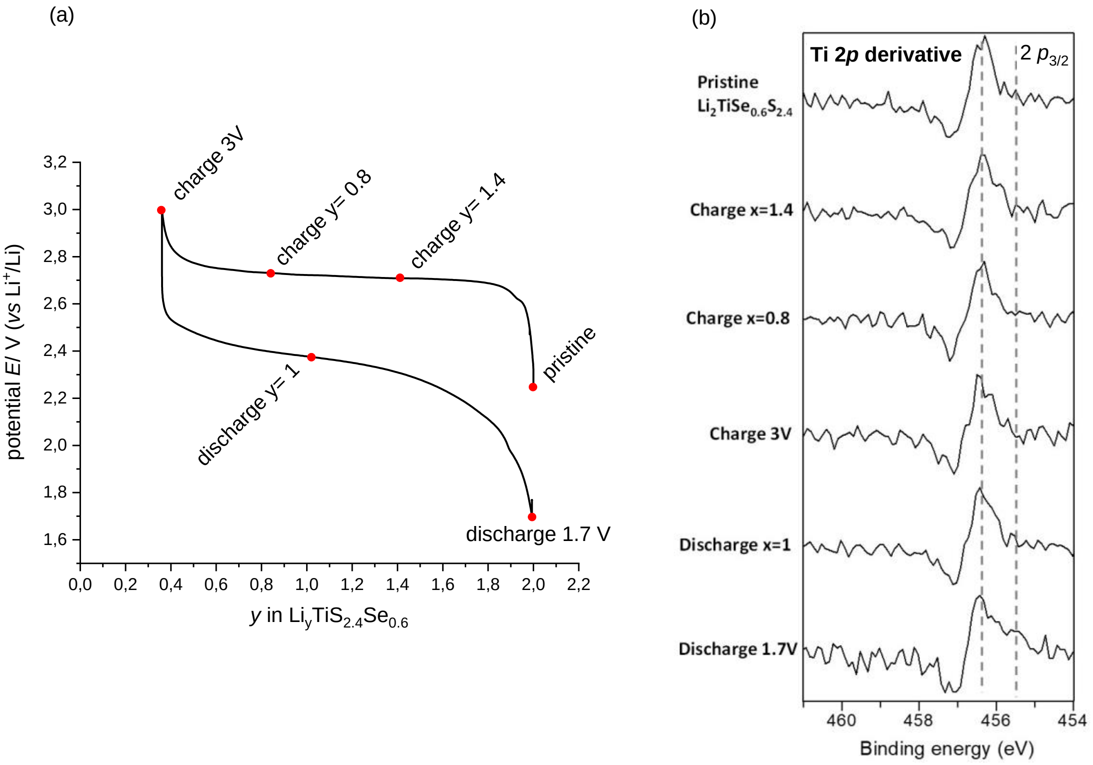

Supplementary Figure 16: HAXPES experiments on $Li_yTis_{2.4}Se_{0.6}$. (a) First cycle charge-discharge voltage profile with points indicating different states of charge (SOC) at which HAXPES spectra were recorded.(b) Derivative curve of HAXPES Ti $2p_{3/2}$ spectra of $Li_2TiS_{2.4}Se_{0.6}$ at various states of charge, allowing to highlight the appearance of Ti^{3+} at the end of discharge.

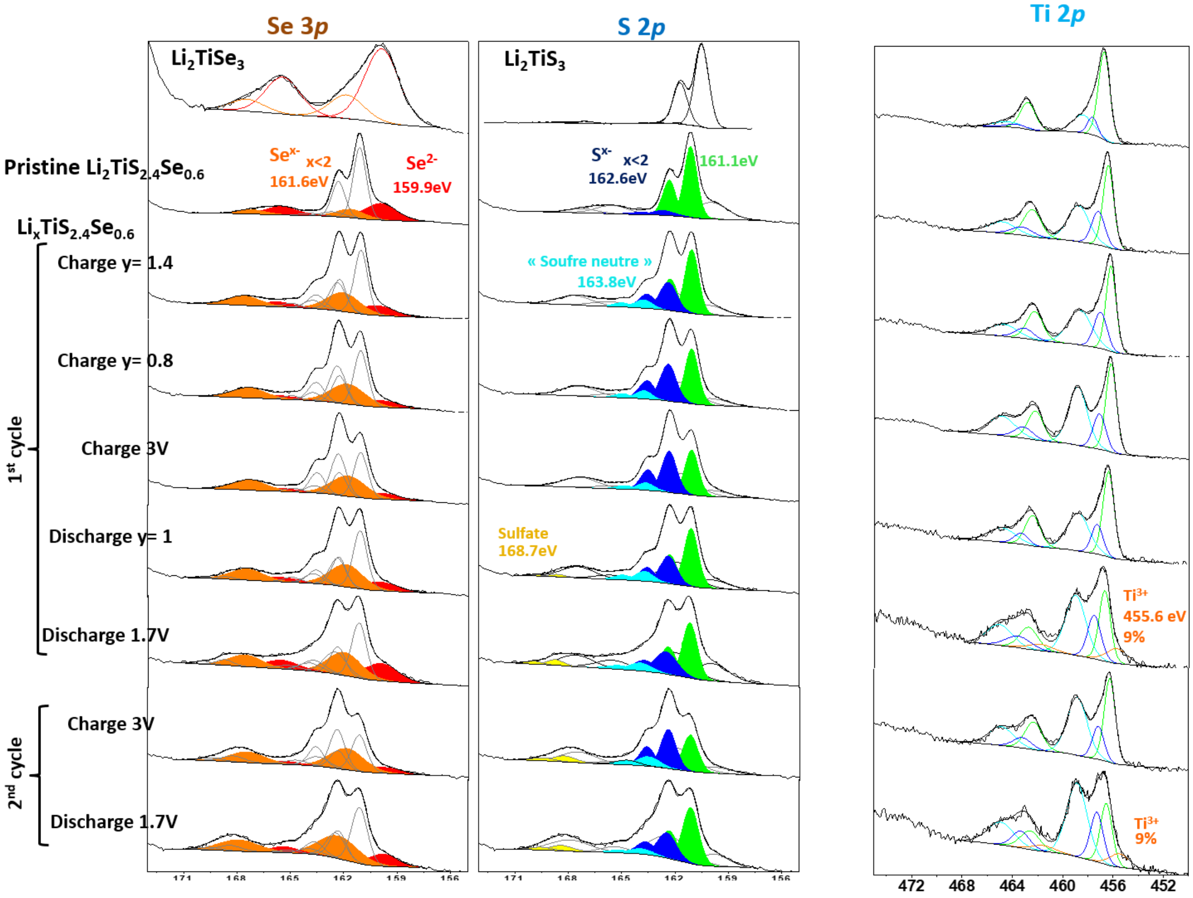

Supplementary Figure 17: XPS data on $Li_2TiS_{2.4}Se_{0.6}$ at various states of charge. The amount of Ti^{3+} in discharged $Li_2TiS_{2.4}Se_{0.6}$ does not change between cycles 1 and 2. The intensity of Ti-O species is considerably higher than in the corresponding HAXPES spectra.

EPR samples

Solution based chemical oxidation and reduction

In order to not contaminate EPR samples with carbon, we explored routes towards chemical charge and discharge of $Li_2TiS_{2.4}Se_{0.6}$, *i.e.* chemical delithiation and relithiation routes to mimic electrochemical cycling.

Chemical delithiation and re-lithiation reaction using iodine in acetonitrile and *n*-butyllithium in hexane respectively were carried out according to the following equations:

$$Li_2TiS_{2.4}Se_{0.6} + 0.85\, I_2 \rightarrow\ Li_{0.3}TiS_{2.4}Se_{0.6} + 1.7\, LiI\ (in\ acetonitrile, at\ RT, 12h)$$

$$Li_{0.3}TiS_{2.4}Se_{0.6} + 1.7\, n - BuLi \rightarrow Li_2TiS_{2.4}Se_{0.6} + 0.85\, n - octane\ (in\ hexane, at\ RT, 12h)$$

Lattice parameters of pristine, electrochemically delithiated, electrochemically re-lithiated, chemically delithiated and chemically re-lithiated $Li_2TiS_{2.4}Se_{0.6}$ are compared below. Electrochemically and chemically manipulated $Li_2TiS_{2.4}Se_{0.6}$ show the same lattice parameters, indicating excellent agreement.

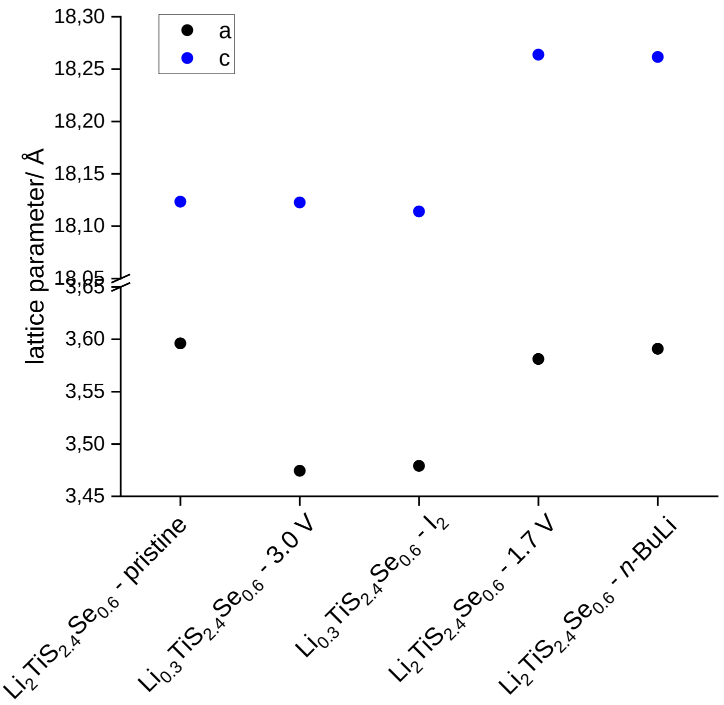

Supplementary Figure 18: Lattice parameters of electrochemically and chemically modified $Li_2TiS_{2.4}Se_{0.5}$ (refined in the $R\bar{3}m$ space group).

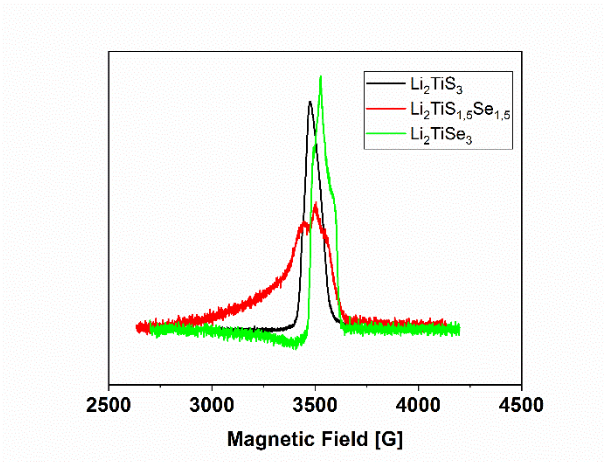

Supplementary Figure 19: EPR spectra of pristine Li_2TiS_3, $Li_2TiS_{1.5}Se_{1.5}$ and Li_2TiSe_3 measured at 5 K.

Solid state NMR

The room temperature ^{77}Se NMR spectrum is given in Supplementary Figure 20 (a) and can satisfactorily be fitted with 5 lorentzian peaks centred at 1061, 1093, 1140, 1185 and 1235 ppm with proportions 19%: 2%: 20%: 24%: 36% (see Supplementary Table 6 for the detailed parameters of the fit). These five environments correspond to slightly different Se environments in the structure, slightly distorted depending on the number of Li and Ti in the first coordination sphere. Assuming full cation disorder in the mixed Li-Ti layer, only 4 different coordination spheres around Se are expected in proportions 4%:22%:43%:29%, suggesting stacking faults or an amorphous impurity.

From the X-ray structure; four different coordination spheres around Se are conceivable $SeLi_3Li_kTi_{3-k}$, with k ranging from 0 to 3: three positions are occupied by Li atoms in the Li layers and three are randomly occupied by Li or Ti from the mixed Li-Ti layer, with occupancies of 0.33 and 0.66 respectively. Assuming fully random cation disorder, the probabilities of these four environments would be $C_3^k \times 0.33^k \times 0.66^{3-k}$ ($SeLi_6$: 4%, $SeLi_5Ti$: 22%, $SeLi_4Ti_2$: 43%, $SeLi_3Ti_3$: 29%). Detailed assignment necessitates chemical shift simulations beyond the scope of the current report.

Turning to the ^7Li NMR spectrum measured at 62.5 kHz MAS, it consists of three peaks at -1.0 ppm (65%) with two secondary lithium local environments at 1.0 ppm (13%) and -0.8 ppm (23%), see Supplementary Table 7 for fitting parameters. Here again, the fully random structure predicts only two lithium sites, so that a lithium-containing impurity phase or stacking faults are expected.

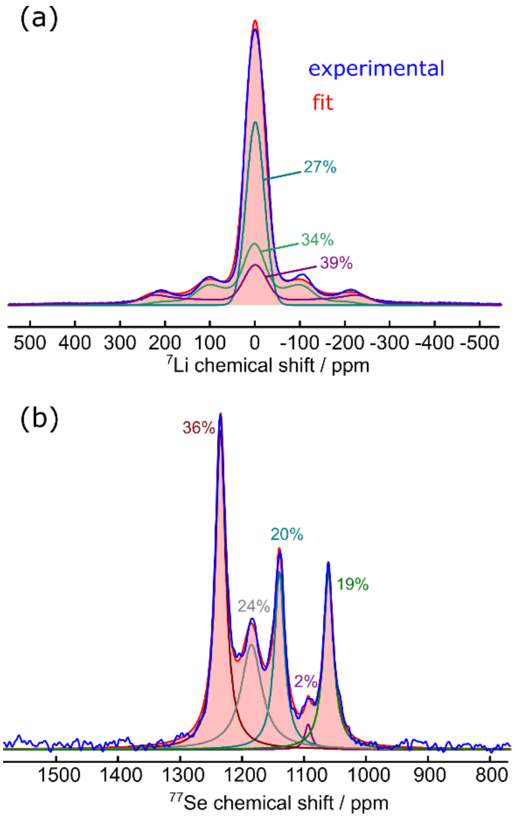

Supplementary Figure 20: NMR spectra of pristine Li_2TiSe_3 at 268 K. (a) ^{77}Se NMR spectrum (b) ^7Li static NMR spectrum.

Supplementary Table 6: Parameters for the ^{77}Se NMR fit.

Model	Peak shift/ ppm	Width/ ppm	%
Lorentzian	1061	19	19
Lorentzian	1093	13	2
Lorentzian	1140	20	20
Lorentzian	1185	40	24
Lorentzian	1235	20	36

Supplementary Table 7: Parameters for the ^{7}Li MAS-NMR fit.

Model	Peak shift/ ppm	Width/ ppm	%
Lorentzian	-1.0	1.8	22.8
Gaussian	-0.8	0.7	64.6
Gaussian	1.0	2.2	12.5

Supplementary Table 8: Longitudinal relaxation rates of ^{7}Li obtained in NMR experiments for temperatures ranging from 118 K to 292 K. The limited amount of time available at the lowest temperatures results in low S/N ratios. No more than 2 components could be reliably constrained.

T/ K	R_1a/ s^{-1}	R_1b/ s^{-1}
292	0.22	0.049
268	0.30	0.075
263	0.26	0.063
223	0.36	0.065
181	0.56	0.073
138	2.24	0.143
118	2.49	0.185

Supplementary Table 9: Parameters for the ^{7}Li static NMR fit at -5°C. The quadrupolar asymmetry was set to zero.

Model	peak shift/ ppm	Width/ ppm	Quadrupolar frequency ν_Q/ kHz	%
Lorentzian	-1.0	48	n.a.	38.8
Quadrupolar 1st order	-0.8	58 (Lorentzian)	38	27.3
Quadrupolar 1st order	1.0	53 (Lorentzian)	18	34.0

For the temperature dependent experiments, a static measuring mode was employed since Magic Angle Spinning is not feasible in such low temperature range. The static ^{7}Li NMR spectra were fitted with 3 independent peaks. Peaks 1 and 2 with a quadrupolar model calculated to 1st order, asymmetry parameter fixed to 0, chemical shifts fixed to 1.0 ppm and -0.8 ppm, respectively. Peak 3 was modelled with a Gaussian peak, with the chemical shift and width left free:

Supplementary Table 10: Parameters for the ^{7}Li static NMR fits

T / K	297	292	268	263	223	181	138	118
Width peak 1 /ppm	-55	-56	-53	-46	-48	-58	-58	-58
Width peak 2 /ppm	-61	-60	-58	-61	-64	-67	-66	-66
ν_Q peak 1 /kHz	15	16	18	18	16	17	17	17
ν_Q peak 2 /kHz	38	38	38	38	37	40	39	39
Chemical shift peak 3 /ppm	-1.0	-1.0	-1.0	-1.0	-1.0	1.7	5.6	5.8
Width peak 3 /ppm	53	53	47	48	58	57	60	62
% peak 1	34.0	33.3	34.0	34.7	33.2	32.9	32.6	33.1
% peak 2	18.7	19.9	27.3	27.5	14.2	18.6	21.1	14.2
% peak 3	47.3	46.7	38.8	37.7	52.7	48.5	46.3	52.7

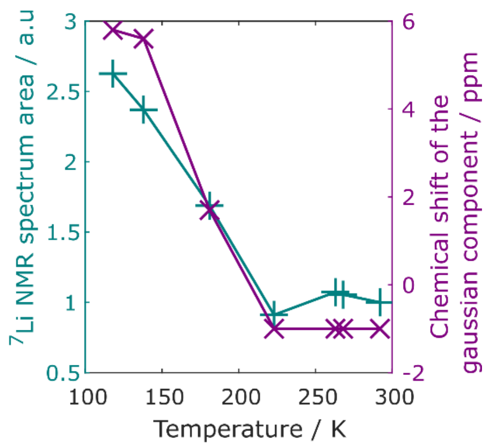

Supplementary Figure 21: area (green) and chemical shift (purple) of the ^{7}Li signal plotted against the temperature.

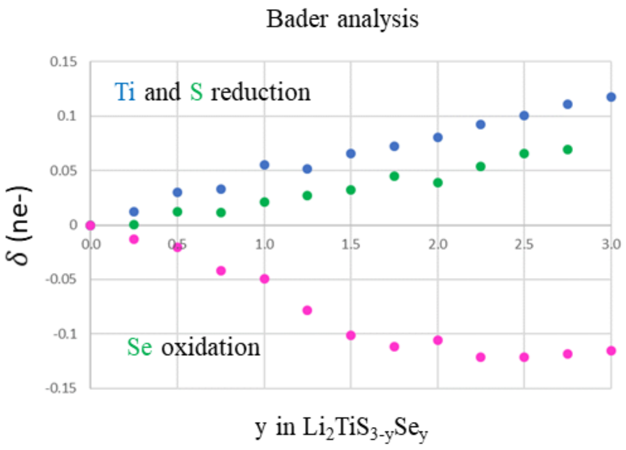

Supplementary Figure 22: Bader analysis of $Li_2TiS_{3-x}Se_x$: Variation of the averaged net population of Ti (blue), S (green) and Se (pink) as a function of the Se content (x) with respect to their reference value in Li_2TiS_3. The δ value are obtained from the atomic Bader analysis performed on various S/Se compositions with the metaGGA SCAN functional. In such calculations, positive and negative δ stand for an atomic reduction and oxidation, respectively. The results are in very good agreement with XPS and EPR measurements showing that the increase of Se in the materials correlates with an increase of the Se^{n-} - species and an increase of Ti^{3+}, respectively. Interestingly, the bell-shape behavior observed in the electrochemical capacity of the $Li_{2-y}TiS_{3-x}Se_x$ electrodes is also seen in the oxidation degree of Se.

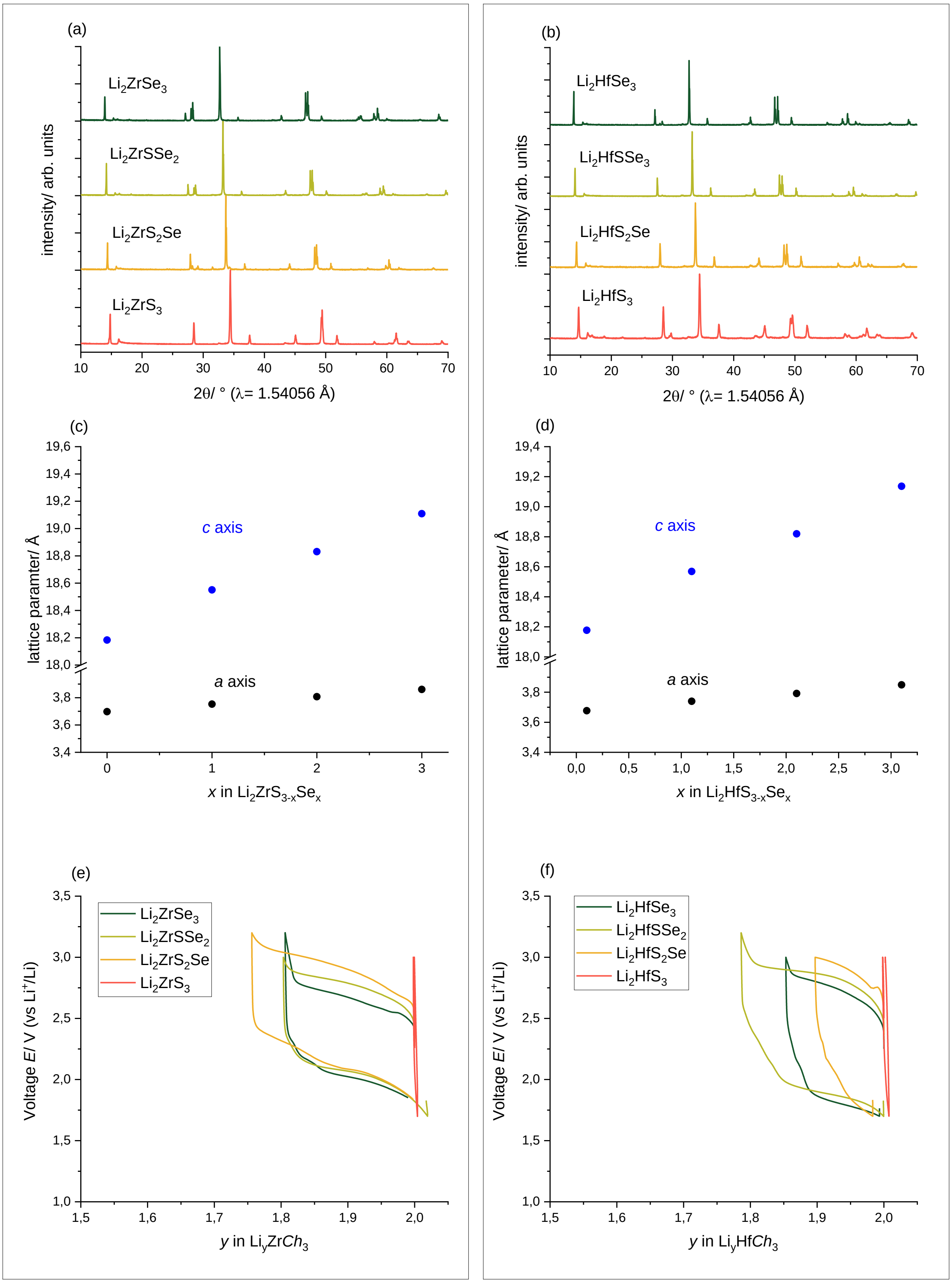

Supplementary Figure 23: Overview of $Li_2S_{3-x}Se_x$ (M= Zr, Hf). (a) + (b): PXRD patterns. (c) + (d): lattice parameters refined in the $R\overline{3}m$ space group. (e) + (f): galvanostatic cycling against Li^+/Li.

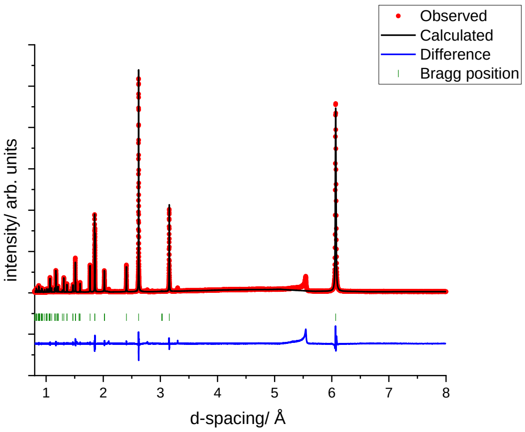

Supplementary Figure 24: Rietveld refinement of Li_2ZrS_3 in the $R\overline{3}m$ space group.

Supplementary Table 11: Crystallographic data of the Rietveld refinement of Li_2ZrS_3 in the $R\overline{3}m$ space group (a= 3.699762(4) Å, c= 18.20667(4) Å, χ^2(SXRD)= 3.31).

Atom	Wyckoff site	x	y	z	occupancy	U_{iso}/ Å^2
S1	6*c*	0.0	0.0	0.25266(3)	1.0	0.01368(19)
Zr1	3*a*	0.0	0.0	0.0	0.66667	0.00726(9)
Li1	3*a*	0.0	0.0	0.0	0.33333	0.00726(9)
Li2	3*b*	0.0	0.0	0.5	1.0	0.0248(18)

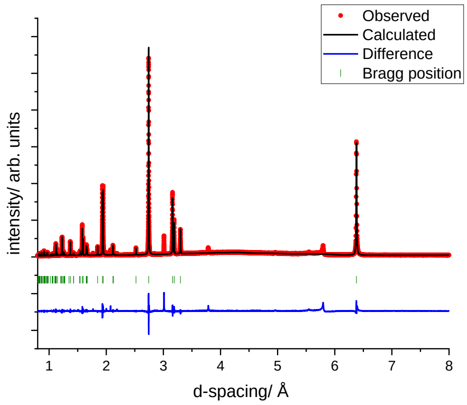

Supplementary Figure 25: Rietveld refinement of Li_2ZrSe_3 in the $R\bar{3}m$ space group.

Supplementary Table 12: Crystallographic data of the Rietveld refinement of Li_2ZrSe_3 in the $R\bar{3}m$ space group (a= 3.866533(5) Å, c= 19.13937(4) Å, χ^2(SXRD)= 3.36).

Atom	Wyckoff site	x	y	z	occupancy	U_{iso}/ Å^2
Se1	6*c*	0.0	0.0	0.25225(2)	1.0	0.01500(9)
Zr1	3*a*	0.0	0.0	0.0	0.66667	0.00886(15)
Li1	3*a*	0.0	0.0	0.0	0.33333	0.00886(15)
Li2	3*b*	0.0	0.0	0.5	1.0	0.034(3)

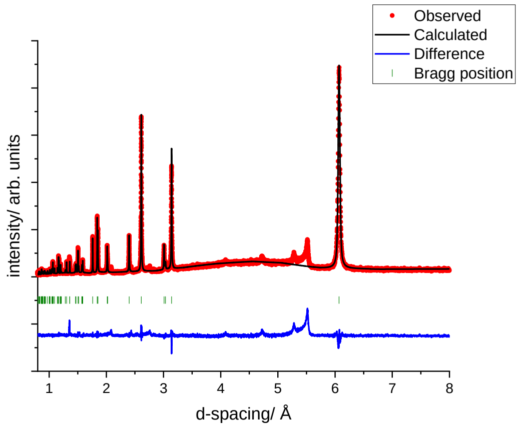

Supplementary Figure 26: Rietveld refinement of Li_2HfS_3 in the $R\overline{3}m$ space group.

Supplementary Table 13: Crystallographic data of the Rietveld refinement of Li_2HfS_3 in the $R\overline{3}m$ space group (a= 3.681710(15) Å, c= 18.20911(14) Å, χ^2(SXRD)= 2.47).

Atom	Wyckoff site	x	y	z	occupancy	U_{iso}/ Å^2
S1	6*c*	0.0	0.0	0.25320(8)	1.0	0.0070(3)
Hf1	3*a*	0.0	0.0	0.0	0.66667	0.00457(9)
Li1	3*a*	0.0	0.0	0.0	0.33333	0.00457(9)
Li2	3*b*	0.0	0.0	0.5	1.0	0.070(6)

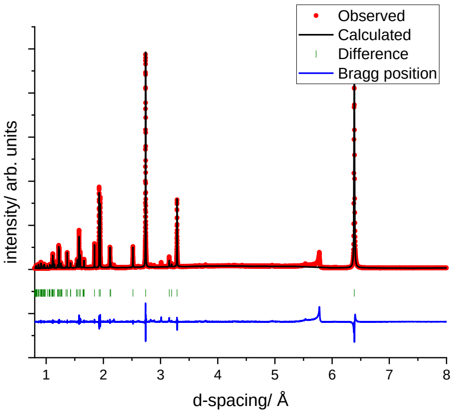

Supplementary Figure 27: Rietveld refinement of Li_2HfSe_3 in the $R\overline{3}m$ space group.

Supplementary Table 14 : Crystallographic data of the Rietveld refinement of Li_2HfSe_3 in the $R\overline{3}m$ space group (a= 3.854201(3) Å, c= 19.16223(3) Å, χ^2(SXRD)= 4.59).

Atom	Wyckoff site	x	y	z	occupancy	U_{iso}/ $Å^2$
Se1	6*c*	0.0	0.0	0.25286(2)	1.0	0.01145(12)
Hf1	3*a*	0.0	0.0	0.0	0.66667	0.0126651(3)
Li1	3*a*	0.0	0.0	0.0	0.33333	0.0126651(3)
Li2	3*b*	0.0	0.0	0.5	1.0	0.069(5)

χ_{Ti} = 1.54 (Pauling) ; r(Ti^{4+}) = 0.745 Å

χ_{Zr} = 1.33 (Pauling) ; r(Zr^{4+}) = 0.86 Å

χ_{Hf} = 1.30 (Pauling) ; r(Hf^{4+}) = 0.85 Å

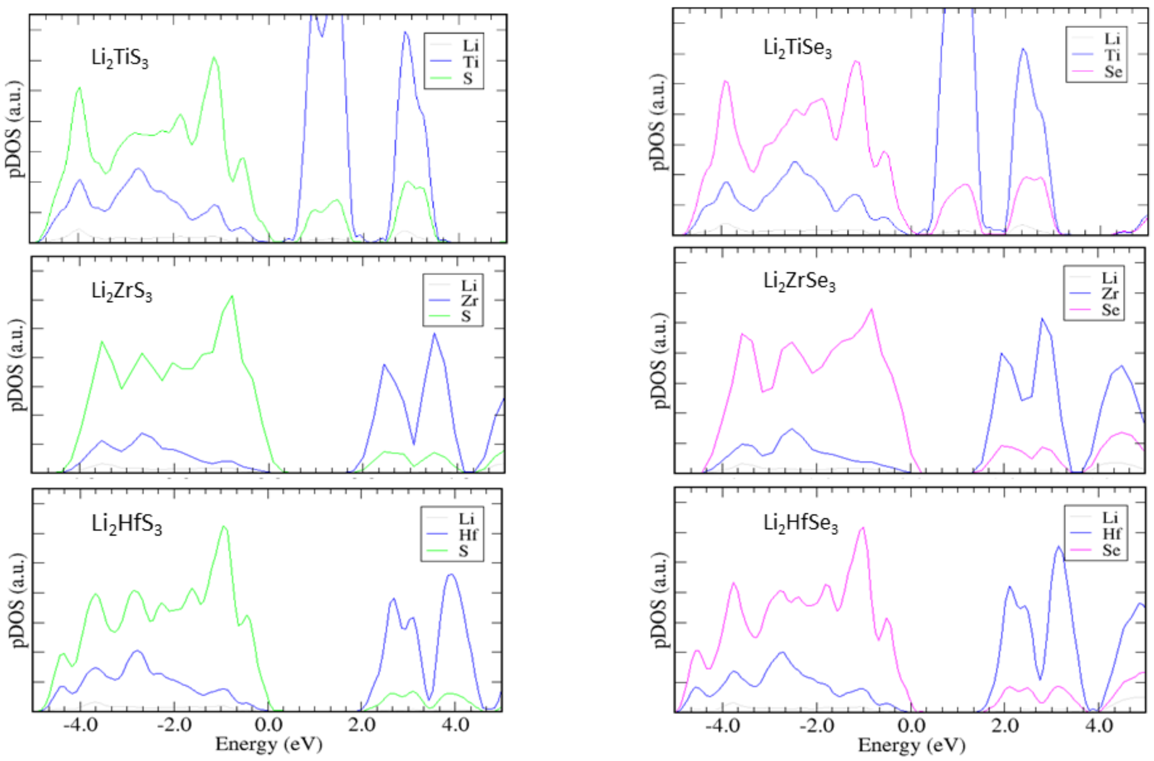

Supplementary Figure 28: Atom-projected Density Of States (pDOS) for sulfides (left) and selenides (right) Li_2MCh_3 (M = Ti, Zr, Hf) computed with the metaGGA SCAN functional and showing the relative positioning of the anionic and cationic bands with respect to the electronegativity difference (Delta Chi) of the transition metal and chalcogen. As expected, the smaller Delta Chi, the closer the anionic and cationic bands.

Supplementary References

(1) Kresse, G.; Furthmuller, J. Efficiency of Ab-Initio Total Energy Calculations for Metals and Semiconductors Using a Plane-Wave Basis Set. *Comput. Mater. Sci.* **1996**, *6* (1), 15–50. https://doi.org/Doi 10.1016/0927-0256(96)00008-0.

(2) Kresse, G.; Joubert, D. From Ultrasoft Pseudopotentials to the Projector Augmented-Wave Method. *Phys. Rev. B* **1999**, *59* (3), 1758–1775. https://doi.org/DOI 10.1103/PhysRevB.59.1758.

(3) Blöchl, P. E. Projector Augmented-Wave Method. *Phys. Rev. B* **1994**, *50* (24), 17953–17979. https://doi.org/10.1103/PhysRevB.50.17953.

(4) Perdew, J. P.; Burke, K.; Ernzerhof, M. Generalized Gradient Approximation Made Simple. *Phys. Rev. Lett.* **1996**, *77* (18), 3865–3868. https://doi.org/DOI 10.1103/PhysRevLett.77.3865.

(5) Dudarev, S. L.; Botton, G. A.; Savrasov, S. Y.; Humphreys, C. J.; Sutton, A. P. Electron-Energy-Loss Spectra and the Structural Stability of Nickel Oxide: An LSDA+U Study. *Phys. Rev. B* **1998**, *57* (3), 1505–1509. https://doi.org/10.1103/PhysRevB.57.1505.

(6) Parr, R. G.; Yang, W. Density Functional Approach to the Frontier-Electron Theory of Chemical Reactivity. *J. Am. Chem. Soc.* **1984**, *106* (14), 4049–4050. https://doi.org/10.1021/ja00326a036.

(7) Massiot, D.; Fayon, F.; Capron, M.; King, I.; Le Calvé, S.; Alonso, B.; Durand, J.-O.; Bujoli, B.; Gan, Z.; Hoatson, G. Modelling One- and Two-Dimensional Solid-State NMR Spectra. *Magn. Reson. Chem.* **2002**, *40* (1), 70–76. https://doi.org/https://doi.org/10.1002/mrc.984.
